# Proteomic Profiling of Small Extracellular Vesicles Secreted by Human Pancreatic Cancer Cells Implicated in Cellular Transformation

**DOI:** 10.1038/s41598-020-64718-6

**Published:** 2020-05-07

**Authors:** Kelly A. Servage, Karoliina Stefanius, Hillery Fields Gray, Kim Orth

**Affiliations:** 1 Department of Molecular Biology, University of Texas Southwestern Medical Center, Dallas, TX USA; 2 Howard Hughes Medical Institute, University of Texas Southwestern Medical Center, Dallas, TX USA

**Keywords:** Mass spectrometry, Cancer microenvironment

## Abstract

Extracellular vesicles secreted from tumor cells are functional vehicles capable of contributing to intercellular communication and metastasis. A growing number of studies have focused on elucidating the role that tumor-derived extracellular vesicles play in spreading pancreatic cancer to other organs, due to the highly metastatic nature of the disease. We recently showed that small extracellular vesicles secreted from pancreatic cancer cells could initiate malignant transformation of healthy cells. Here, we analyzed the protein cargo contained within these vesicles using mass spectrometry-based proteomics to better understand their makeup and biological characteristics. Three different human pancreatic cancer cell lines were compared to normal pancreatic epithelial cells revealing distinct differences in protein cargo between cancer and normal vesicles. Vesicles from cancer cells contain an enrichment of proteins that function in the endosomal compartment of cells responsible for vesicle formation and secretion in addition to proteins that have been shown to contribute to oncogenic cell transformation. Conversely, vesicles from normal pancreatic cells were shown to be enriched for immune response proteins. Collectively, results contribute to what we know about the cargo contained within or excluded from cancer cell-derived extracellular vesicles, supporting their role in biological processes including metastasis and cancer progression.

## Introduction

Small extracellular vesicles (sEVs) are one type of secreted vesicle, ranging in size from 30–150 nm, that are naturally produced and released from all eukaryotic cells^1–3^. These small vesicles are either formed in the endosomal compartment of cells and released after fusion of the multivesicular body (MVB) with the plasma membrane of cells or can be derived directly from the plasma membrane^4^. Before release, sEVs are packaged with important biological cargo including, proteins, DNA, microRNA, lipids, and metabolites, and have been shown to facilitate cell-cell communication^2,5–10^. Because sEVs are routinely secreted by tumor cells into the tumor microenvironment, there is growing interest in the field in investigating their role in cell proliferation, metastasis, and cancer progression^11–19^.

Pancreatic cancer is a highly metastatic cancer and one of the leading causes of cancer-related deaths due to late detection rates and lack of effective treatments^20^. Recent studies have advanced our understanding of pancreatic cancer through the discovery of different driver mutations in pre-malignant lesions surrounding the primary pancreatic tumor site, suggesting that not all tumor cells are derived from clonal events^21–23^. Vesicles secreted from cancer cells may be implicated in these independent transformation events in the tumor microenvironment, as pancreatic cancer cell sEVs have been shown to play a crucial role in metastasis of the cancer, specifically by prompting pre-metastatic niche formation in the liver^13^. Our recent work showed that sEVs isolated from pancreatic cancer cells contribute to malignant cell transformation by functioning as an initiator of transformation in a classic two-stage assay^24^. Malignant cell transformation *in vitro* was induced by exposing NIH/3T3 cells to a two-step treatment by an initiator and then a promoter^25,26^. Classic initiators are typically suspected carcinogens that manipulate the recipient cells upon treatment by incorporating random genetic mutations to cells. Subsequent treatment of these mutated cells with a promoter, like the drug TPA (12-O-tetradecanoylphorbol 13-acetate), will enhance cell proliferation and drive malignant cell transformation^25^. Our previous work revealed a distinct difference in the role that pancreatic cancer cell sEVs and normal pancreatic cell sEVs play in malignant cell transformation. Isolated sEVs from multiple types of pancreatic cancer cells could successfully function as an initiator in this assay and lead to malignant cell transformation. Additionally, these transformed cells were shown to be tumorigenic *in vivo*. This initiator capability, however, was found to be unique to sEVs secreted from cancer cells and not those secreted from normal pancreatic epithelial cells. While the mechanism of how these cancer cell sEVs are manipulating recipient cells is still not fully understood, it is clear that there are distinct differences between sEVs secreted from cancer and normal pancreatic cells in this context.

Considering that it is still not clear why or even whether certain proteins are selectively packaged into different types of EVs in cells, this study aims to gain a better understanding of this process for both cancer and normal pancreatic cells. Here, we carried out an in-depth proteomic analysis on four types of pancreatic cell sEVs that were used in our aforementioned study^24^. Three different pancreatic cancer cell sEVs (Capan-2, MIA PaCa-2, and Panc-1) were compared to sEVs isolated from normal human pancreatic ductal epithelial cells (HPDE). By using a mass spectrometry (MS)-based proteomics approach, we were able to elucidate differences in the protein cargo of sEVs secreted from different types of pancreatic cells and analyze those differences based on related biological functions. Ultimately, a small group of proteins are found in common between all types of cancer sEVs studied that were not identified in normal HPDE sEVs. These proteins are largely involved in processes pertaining to the formation and trafficking of vesicles in the endosomal system of cells. They also include a set of proteins that have been previously implicated in malignant cell transformation. Conversely, there are a number of immune response proteins identified in sEVs secreted from normal, healthy pancreatic cells that are not found in any of the pancreatic cancer cell sEVs. These differences in the proteomes of cancer and normal sEVs shown here may be indicative of their varying roles in cell transformation and helpful in delineating the types of EVs that are being produced.

## Results and discussion

### Characterization of isolated sEVs from pancreatic cells

**Figure 1:**
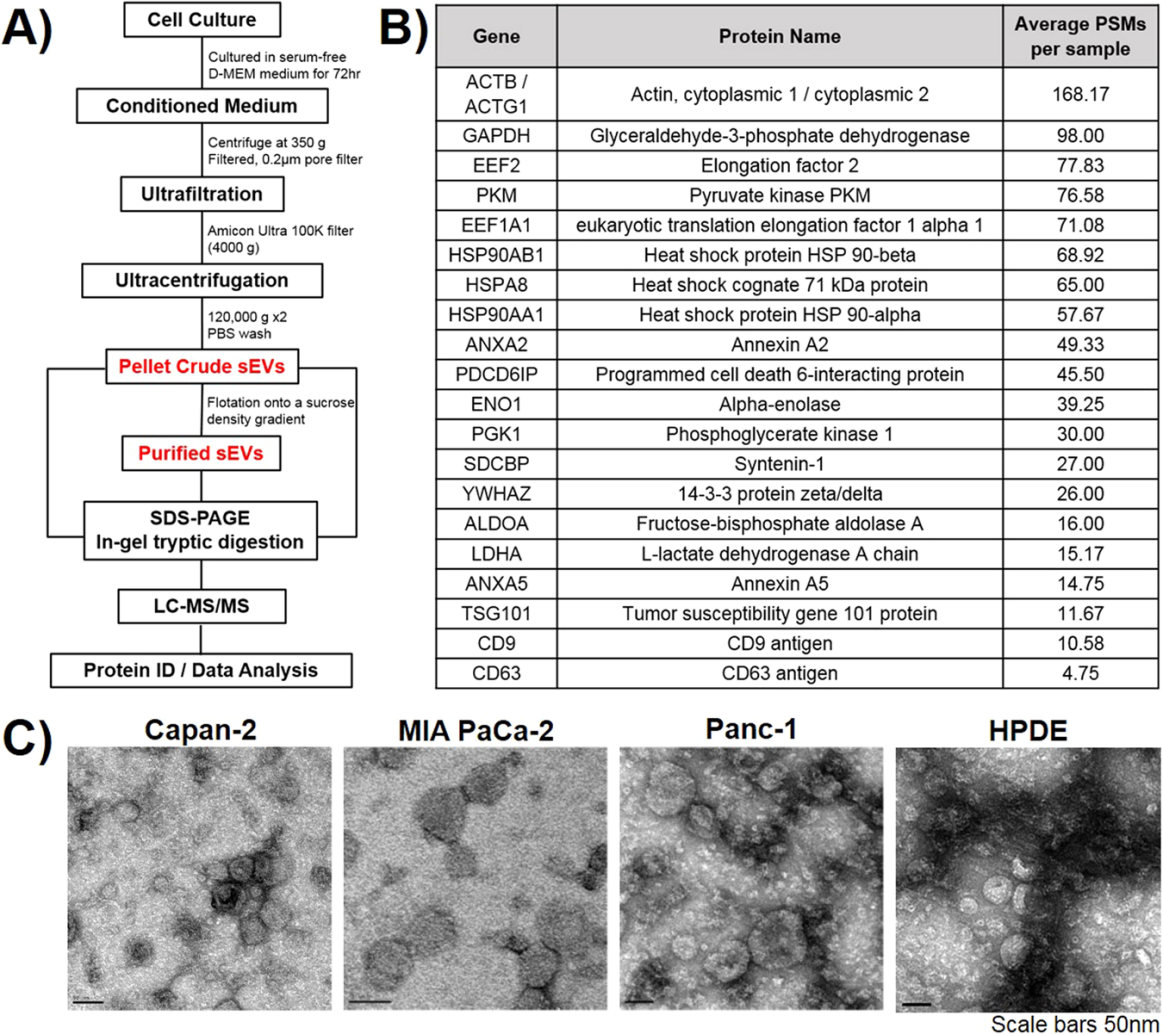
Isolation and characterization of small extracellular vesicles (sEVs) from four human pancreatic cell lines: Capan-2, MIA PaCa-2, Panc-1, and HPDE. **(A)** Isolation protocol and workflow for LC-MS/MS analysis of sEVs. Detailed protocol included in Methods. **(B)** Table of common sEV marker proteins found by MS: average peptide spectral matches (PSMs) were calculated from three biological replicates for each of the four types of sEVs. **(C)** TEM images of Capan-2, MIA PaCa-2, Panc-1, and HPDE sEVs (data reprinted from ref. ^24^).

To assess the proteomes of the four types of pancreatic sEVs, we performed proteomics experiments using liquid chromatography-tandem mass spectrometry (LC-MS/MS). Three types of cancer cell sEVs that were previously shown to function as an initiator of cell transformation were analyzed: Capan-2, MIA PaCa-2, and Panc-1, in addition to sEVs from one normal pancreatic cell line (HPDE). All vesicles were isolated using a combined ultrafiltration-ultracentrifugation method to isolate “crude” sEVs from each cell type (Fig. 1A)^24,27^. Briefly, sEVs were isolated by first removing cells, cellular debris, and larger vesicles by centrifugation and filtration through a 0.2 mm pore filter. Enrichment for sEVs was then achieved by ultrafiltration and ultracentrifugation^24,27^. The resulting crude sEV pellets were normalized based on protein concentration and run on SDS-PAGE gels for LC-MS/MS analysis. Considering that our aim is to analyze the in-depth proteomes of vesicles that previously exhibited initiator activity, it was important to maintain a consistent sEV isolation method with the one previously published^24^. According to guidelines published in the Minimal Information for Studies of Extracellular Vesicles (MISEV2018), the combined ultrafiltration-ultracentrifugation method we used to generate crude sEVs is classified as an “intermediate recovery/intermediate specificity” isolation method^4^. This means there will likely be some contamination of isolated sEVs with aggregated proteins or nucleic acids. More recently, it has become apparent that a combined ultrafiltration-ultracentrifugation method coupled with a sucrose density gradient purification method results in separation of a “more pure” population of sEVs^28^. Therefore, this method was tested with Capan-2 cells for comparison with crude sEVs. For this, crude sEVs were floated onto a sucrose density gradient and the resulting sEV fraction at a density of 1.15–1.174 g/mL (Fraction 3) was analyzed (Fig. 1A, Fig. 6). Using this method, recovery was sacrificed in order to benefit from the enhanced purification of sEVs resulting in a decrease in the number of observed proteins (Fig. 6). When protein concentration of crude and sucrose density gradient separated sEVs is normalized, an enrichment in common sEV markers is observed by Western blot analysis and MS/MS spectral count comparison (**Supplementary** Figure S1).

After isolation, sEVs were characterized according to guidelines laid out in the MISEV2018^4^. Common sEV marker proteins were identified in crude sEVs by MS and were confirmed previously by western blot analysis in addition to western blot analysis of proteins underrepresented in sEVs (**Supplementary** Figure S2)^24^. The top twenty highest occurring proteins found in studies on sEVs (ExoCarta database)^29^ were each identified in our samples (Fig. 1B); the average peptide spectral matches (PSMs) for each protein were calculated by averaging the number of PSMs for three biological replicates of each of the four types of crude sEVs (12 samples total). Additionally, the expected morphology of vesicles was confirmed using electron microscopy (TEM) (Fig. 1C) and the size of crude Capan-2 sEVs, as determined by nanoparticle tracking analysis, was centered on 91 nm or 8.75 × 10^8^ particles/µg (Supplementary Fig. S1); both images have been reprinted with permission from ref. ^24^.

### Comparative proteomic analysis of sEVs isolated from pancreatic cells

**Figure 2:**
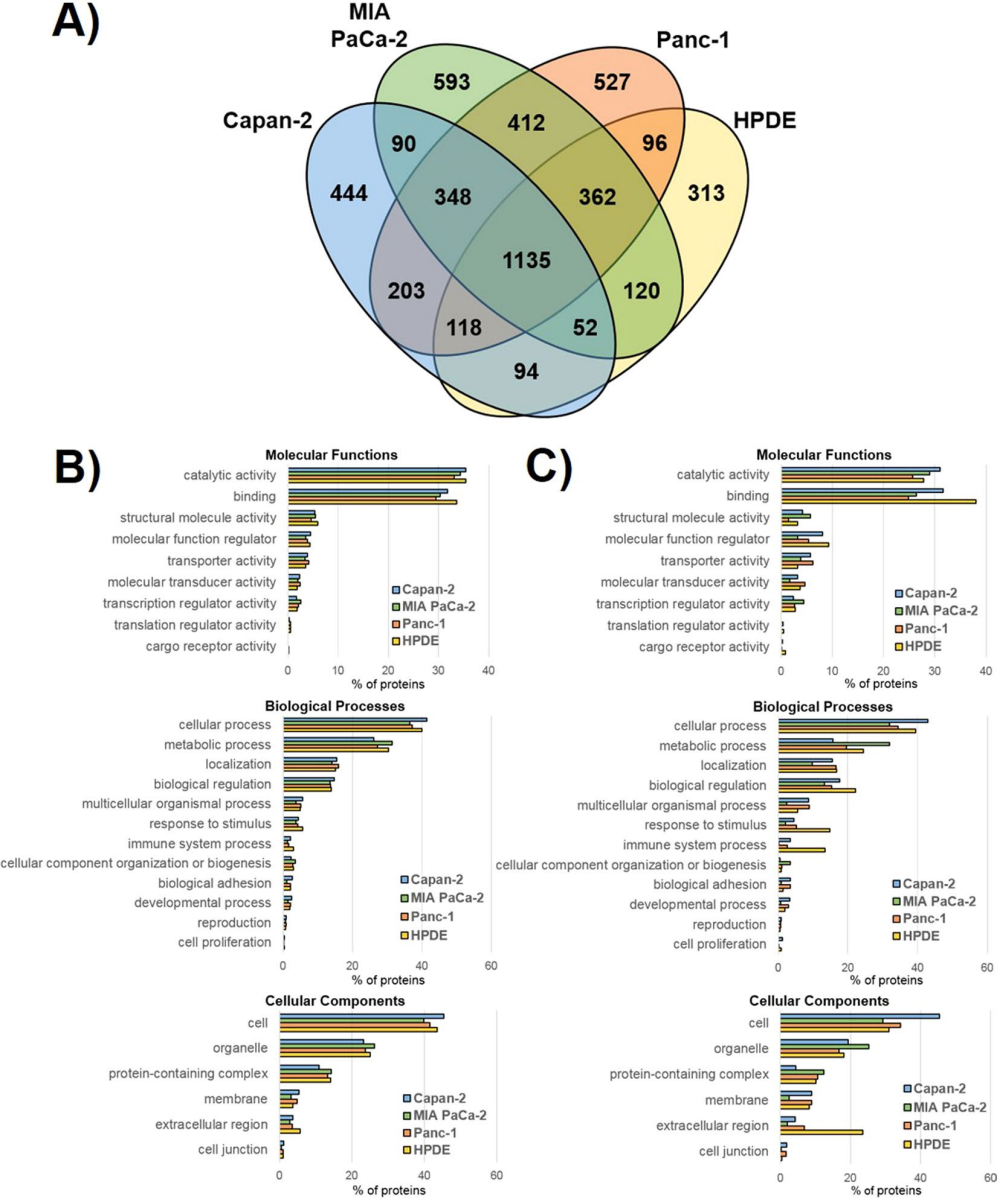
Comparison of proteins found in sEVs from human pancreatic cell lines. **(A)** Venn diagram showing the overlap of proteins found in Capan-2, MIA PaCa-2, Panc-1, and HPDE sEVs, n = 3 for each type of sEV. **(B)** PANTHER classification of the molecular functions, biological processes, and cellular components associated with the complete protein profile of sEVs from each of the four human pancreatic cell lines. **(C)** PANTHER classification of only the unique proteins found in each type of sEV; analysis was performed on 444, 593, 527, and 313 unique proteins found in Capan-2, MIA PaCa-2, Panc-1, and HPDE, respectively.

When the proteomes of the four types of pancreatic sEVs were compared (Fig. 2A), a total of 4,907 unique proteins were identified confidently across the combined samples (peptide FDR < 0.01, protein FDR < 0.01, n = 3 for each cell type). According to the database ExoCarta, over 9,000 total proteins have been identified in studies on vesicles sized 30–150 nm^29^. It is likely, however, that many of the proteins identified previously in sEVs are general housekeeping proteins responsible for basic cellular function. Here, 1,135 of the 4,907 identified sEV proteins were found in all four types of sEVs. When the full protein profile for each sEV type was classified independently based on molecular properties via PANTHER 14.1^30,31^, the distribution of proteins among different categories was consistent between all four types of sEVs (Fig. 2B). However, there were also a number of proteins found to be unique to only one type of sEV: 444 Capan-2 proteins, 593 MIA PaCa-2 proteins, 527 Panc-1 proteins, and 313 HPDE proteins. Western blot analysis of a representative unique protein from these groups for each sEV type, with the exception of HPDE, was performed for further validation. Western blot analysis is shown for representative proteins SARG, ALDH1A1, and CD13 found uniquely in Capan-2, MIA PaCa-2, and Panc-1 sEVs, respectively (**Supplementary** Figure S3). HPDE-specific proteins stanniocalcin and carboxypeptidase could not be detected using their specific antibodies. When the groups of unique proteins identified for each sEV type were clustered by PANTHER individually, differences in molecular properties were more apparent (Fig. 2C). Specifically, the percentage of unique HPDE sEV proteins involved with biological regulation, response to stimulus, and immune system process (biological processes) and found in the extracellular region (cellular component) are enriched relative to the three cancer sEVs. This indicates that a higher number of proteins linked to these processes were uniquely found in normal HPDE vesicles and not in vesicles from cancer cells.

**Figure 3:**
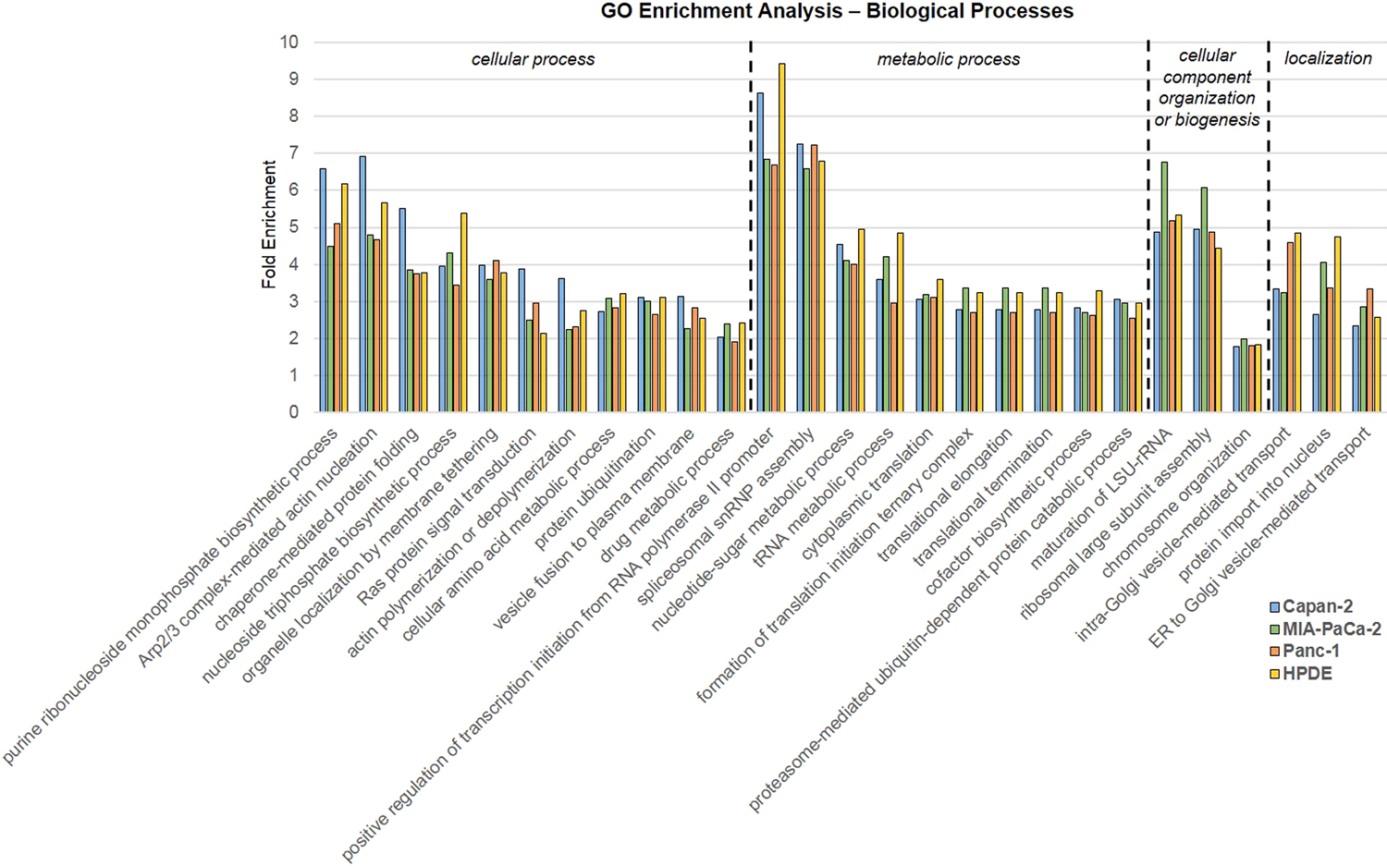
Gene ontology (GO) enrichment analysis on the complete protein profiles for Capan-2, MIA PaCa-2, Panc-1, and HPDE sEVs. Only biological processes found to be significantly enriched in all four types of sEVs are shown. P-values <0.05 were considered as statistically significant. Enriched biological processes are grouped based on parent GO terms including cellular process, metabolic process, cellular component organization or biogenesis, and localization.

Further functional analysis of the four sets of sEV proteins was carried out by performing gene ontology (GO) enrichment analysis on the complete set of proteins for each cell line^30,32,33^. This analysis revealed a set of 27 biological processes that were found to be over-represented consistently in all four types of sEVs, regardless of whether they originated from cancer cells or normal cells (Fig. 3). These enriched processes belong to four parent biological process categories including cellular process, metabolic process, cellular component organization, and localization (Fig. 3). Cellular process categories include general processes that are carried out at the cellular level and are often necessary for cell growth and maintenance. Of the cellular process enriched terms, proteins related to exocytosis, membrane docking, and intracellular signal transduction were all found to be over-represented (Supplementary Table S1). This is consistent with the formation and secretion of sEVs from all cell types and explains why such proteins are over-represented in both cancer and normal cell vesicles. Additionally, proteins associated with cellular metabolic processes, actin filament organization, and protein folding also contributed to the cellular process grouping (Supplementary Table S1). The enriched metabolic process category includes general metabolic processes that contribute to cell growth and a number of functions related to gene expression. The cellular component or biogenesis category includes enriched processes that contribute to the production of molecules within the cell. Lastly, the three processes related to localization are associated specifically with vesicle-mediated transport (Supplementary Table S1). Overall, the biological processes found to be enriched in all four types of sEVs based on their respective protein profiles are either associated with general cell function and growth or related to the endocytic pathway of the vesicles themselves. Thus, it is not surprising that proteins with these general functions are found in vesicles from both cancer and normal cells.

### Comparative proteomic analysis of cancer cell versus normal cell sEVs

**Figure 4:**
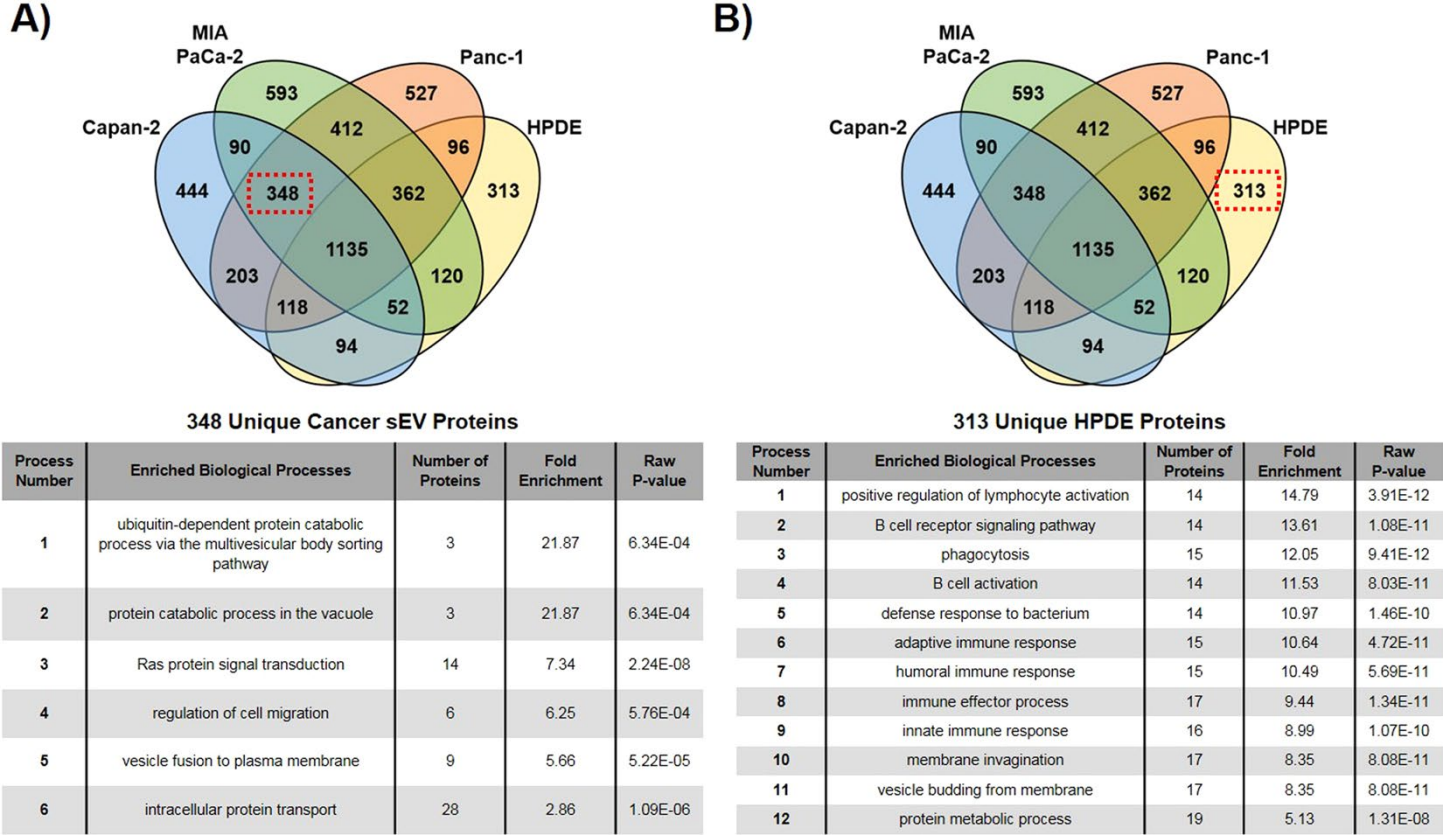
Comparative analysis of unique cancer versus unique normal sEV proteins. **(A)** GO enrichment analysis of 348 common pancreatic cancer sEV proteins that are not found in normal HPDE sEVs. Enriched biological processes found are labeled as “Cancer Processes 1–6.” **(B)** GO enrichment analysis of 313 unique HPDE proteins that are not found in the three pancreatic cancer sEVs. Enriched biological processes found are labeled as “Normal Processes 1–12”.

Although proteomic analysis reveals a number of cell processes in common between cancer and normal cell sEVs, there are also important distinctions. Because our previous work showed that sEVs isolated from different pancreatic cancer cells, but not normal cells, could each contribute to malignant cell transformation by functioning as an initiator, it is of interest to identify differences in proteins found in the three cancer sEVs versus proteins found in normal HPDE sEVs. By comparing the three cancer cell sEVs (Capan-2, MIA PaCa-2, and Panc-1), we found 1,483 shared proteins (Fig. 2A). Among these 1,483 common cancer sEV proteins, 1,135 were also found in normal HPDE sEVs. This means that 348 proteins are identified in all three cancer cell sEVs but not found in the normal HPDE sEVs (Fig. 2A). Western blot analysis of a representative protein from this group (RhoB) confirmed its presence in all three cancer cell sEVs but not normal HPDE sEVs (**Supplementary** Figure S3). To understand the biological properties of these 348 cancer sEV proteins, GO enrichment analysis was performed revealing six enriched biological processes, labeled Processes 1–6 in the table shown in Fig. 4A.

*Cancer Processes 1 and 2*. The two processes with the highest enrichment scores, (1) ubiquitin-dependent protein catabolic process via the MVB sorting pathway and (2) protein catabolic process in the vacuole, each contain the same three contributing proteins (VPS36, SNF8, and VPS37C) (Supplementary Table S2). These proteins are components of the endosomal sorting complex (ESCRT) that regulates sorting of ubiquitinated cargo in the endosome into MVBs^34^. MVBs can either transport cargo like misfolded proteins to the lysosome for degradation or fuse with the plasma membrane resulting in sEV secretion^34,35^.

*Cancer Process 3*. Additionally, there is an over-representation of proteins associated with (3) Ras protein signal transduction among the group of 348 (Fig. 4A). Of the 14 specific proteins contributing to this enrichment, ten Rab proteins were found (Supplementary Table S2). These are Ras-related small GTPases typically found in the endosome or plasma membrane of cells^36^. These proteins are responsible for vesicle formation in the endosome and ultimate transport and secretion of vesicles after fusion of the MVB with the plasma membrane^36,37^. As shown in Fig. 3, enrichment in Ras protein signal transduction was also observed in normal HPDE sEV proteins when the total proteomes were analyzed, but the enrichment score for HPDE was less than that of Capan-2, MIA PaCa-2, and Panc-1. While the presence of Rab proteins in both cancer and normal pancreatic sEVs is expected since they contribute to vesicle formation^36^, our data suggests that these proteins are enriched in cancer cells relative to normal cells. This is likely due to the fact that cancer cells have been shown to produce and secrete more EVs than healthy cells^38,39^. A recent study demonstrated that RAB27B, one of the proteins identified here, is critical for secretion of sEVs from HeLa cells as inhibition of this protein strongly decreased vesicle secretion^37^. Thus, the increased secretion of sEVs from cancer cells also supports the over-representation of ESCRT proteins observed here, since these proteins are also required for vesicle formation^35^. In addition to Rab proteins, GNA12, GNA13, RhoF, and RhoB were also found uniquely in all three cancer cell sEVs and contribute to the enrichment of Ras protein signal transduction (Supplementary Table S2). GNA12 and GNA13 are members of the G12 family of G proteins. Their presence in all three cancer cell sEVs is of particular interest because overexpression of these proteins has the ability to cause transformation of fibroblast cells, including NIH/3T3 cells^40–43^. This is consistent with our previous work showing that the three types of cancer cell sEVs analyzed here could each initiate malignant transformation of NIH/3T3 cells^24^. The cell growth promoting activity of GNA12/13 is attributed to the activation of small GTPases (Ras, Rac, Rho, and CDC42) which mediate signaling through multiple pathways including activation of c-Jun N-terminal kinases (JNKs)^44^, ERK^45^, and Rho-dependent focal adhesion complex formation^46^. RhoB, found here, directly effects activation of JNKs, as loss of RhoB prevents phosphorylation and subsequent activation of JNKs^47^. RhoB was also shown to mediate cell death after DNA damage and to have increased activity in cancer cells after treatment with a DNA damaging agent^47–49^. The activation of Rho by GNA12/13 may also play additional roles in cell transformation through their regulation of the cytoskeleton, as RhoF was shown to cause changes to the actin cytoskeleton of cells affecting cell migration^50^.

*Cancer Process 4*. Enrichment of proteins associated with the (4) regulation of cell migration was also observed in the 348 unique cancer cell sEV proteins (Fig. 4A). Not surprisingly, RhoB and RhoF also contribute to this enrichment for the aforementioned reasons (Supplementary Table S2). This group also includes two semaphorin extracellular signaling proteins (SEMA4B and SEMA3C) and their corresponding surface receptor proteins, Plexin-B2 and Plexin-A1, respectively. The binding of semaphorins by plexins was shown to regulate Rho activity^51^. Additionally, Class B Plexins were shown to play a role in invasive growth and cell migration^52^. It is likely that the interplay between the signaling pathways mediated by small GTPases is essential for the observed transformation of NIH/3T3 cells. The presence of both GNA12/13 and the semaphorin proteins in all three cancer cell sEVs, but not normal cell sEVs, supports a model whereby these proteins may be responsible for activation of these small GTPases.

*Cancer Processes 5 and 6*. Lastly, the over-representation of proteins associated with (5) vesicle fusion to plasma membrane and (6) intracellular protein transport in cancer cell sEVs are likely due to the increased secretion of vesicles from cancer cells relative to normal cells (Fig. 4A). Many of the proteins contributing to these two categories are the same Ras-related proteins discussed previously, as well as additional members of the ESCRT machinery (CHMP6 and CHMP4B) which are components of the ESCRT III complex (Supplementary Table S2)^34,35^. CHMP4B (also known as SNF7) has recently been shown to play a role in plasma membrane deformation by assembling into circular filaments that attach to the membrane, distorting it and allowing for membrane cleavage^53^. These enriched processes also include contributions from a number of SNARE proteins that mediate vesicle fusion with target membranes^54,55^, exocyst complex proteins that are involved with vesicle docking on the plasma membrane^56^, and general proteins that are associated with protein trafficking between cell components^57,58^. Additionally, there are multiple proteins present associated with cell surface processes including SNX17 that regulates endosomal recycling of cell surface proteins during endocytosis^59^ and GOLGA7, a membrane protein involved with protein transport from the Golgi to the cell surface^60^. Interestingly, the small GTPase RAB5A and two ESCRT components CHMP4B and SNF8, each found in all cancer cell sEVs but not normal cell sEVs, are all predicted to be required for the vesicle release of proteins CD63, syndecan, and syntenin-1^61^. These proteins are commonly considered vesicle markers and were shown to interact with one another at the cell surface. Syntenin-1 is an adaptor protein that can bind to both transmembrane proteins CD63 and syndecan^61,62^. Both binding interactions were shown to regulate endocytosis and promote sEV biogenesis^61,62^. Additionally, Syntenin-1 binding has been implicated in processes like cell migration and metastasis, as it was shown that syntenin-1 expression was increased in metastatic cell lines^63^. A previous study showed that knockdown of the three proteins found in our dataset (RAB5A, CHMP4B, and SNF8) each significantly reduced release of CD63, syndecan, and syntenin-1 from sEVs^61^.

**Figure 5:**
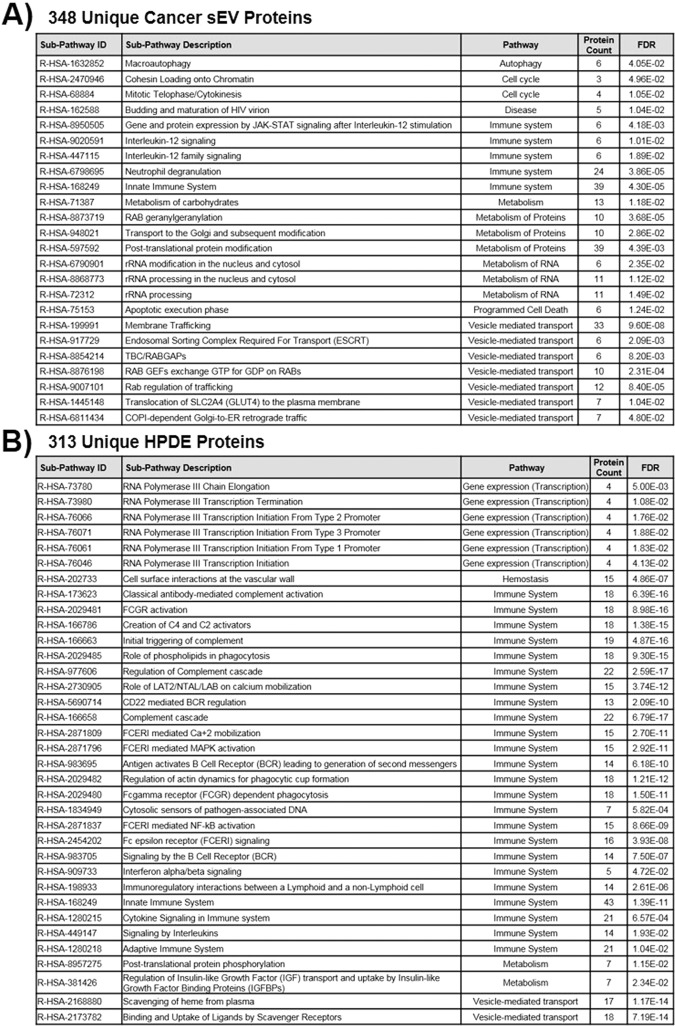
Significantly enriched Reactome Pathways associated with **(A)** the set of 348 common pancreatic cancer sEV proteins and **(B)** the set of 313 unique HPDE proteins from Fig. 4. Enriched Reactome pathways were identified via GO enrichment analysis on the two subsets of proteins.

The 348 unique cancer sEV proteins were also mapped to Reactome pathways and analyzed to determine which pathways were over-represented based on the protein data (Fig. 5, Supplementary Data S1)^64,65^. Analysis was carried out via GO-enrichment and each enriched sub-pathway was grouped based on its corresponding parent Reactome pathway^30^. Results were consistent with the enriched biological processes shown in Fig. 4, as the largest number of over-represented pathways grouped to either vesicle-mediated transport or metabolism. Additionally, pathways related to autophagy, disease, and programmed cell death were also enriched in cancer cell vesicle proteins, indicative of cancer cell origin. Overall, the 348 proteins found in all three cancer cell sEVs appear to be largely enriched for proteins involved in the cellular endosomal system, which encompasses the formation of vesicles, trafficking of different molecules throughout the cell, and ultimate secretion of vesicles to the extracellular matrix. The enrichment of these proteins observed in vesicles from three different pancreatic cancer cell lines may suggest the importance of their role in those processes driving the increased production of sEVs from cancer cells relative to normal healthy cells. Additionally, the presence of a number of proteins that have previously been shown to contribute to oncogenic cell transformation supports our previous finding that pancreatic cancer cell-derived sEVs can initiate malignant cell transformation.

### Characterization of unique proteins identified in normal pancreatic sEVs

Analysis of the 348 proteins found in all three cancer cell sEVs represented a group of proteins absent from normal sEVs, but there were 313 proteins found uniquely in normal HPDE sEVs (Fig. 2A). We analyzed this set of proteins using GO enrichment analysis to determine if there were any enriched processes that were common to normal sEVs but missing from cancer cells (Fig. 4B). Many of the enriched biological processes found for the unique HPDE sEVs were involved with immune response (*Normal Processes 1 to 9*) (Fig. 4B). Among the 313 proteins, there were 14 immunoglobulin proteins that contribute to each of the enriched biological processes listed in Fig. 4B (Supplementary Table S3). Immunoglobulin proteins are membrane-bound or secreted antibodies that are produced from B cells and function as antigen receptors in humoral immunity^66^. Their presence in normal pancreatic epithelial cells is somewhat surprising but explains the increase in extracellular region proteins observed in the PANTHER classification of the unique HPDE proteins shown previously in Fig. 2C. In addition to immunoglobulin proteins, there were also a number of other immune response proteins found in HPDE vesicles that were shown to play roles in differentiation, cell growth, apoptosis, and gene regulation (Supplementary Table S3)^67–72^. Regulation of these processes by immune response proteins may be an important distinction between normal and cancer cells considering that sEVs from normal cells did not contribute to malignant cell transformation^24^. The other biological processes found to be enriched in unique proteins from normal HPDE cell sEVs were membrane invagination, vesicle budding from the membrane, and protein metabolic process (*Normal Processes 10 to 12*) (Fig. 4B). One interesting protein contributing to the enrichment of protein metabolic processes is ITIH1. It is a member of the inter-α trypsin inhibitor family that forms a complex with hyaluronan (HA), the non-sulfated glycosaminoglycan that forms in the plasma membrane of cells and is distributed widely throughout the body^73^. HA has been shown to contribute to processes including cell proliferation, cell migration, and possibly tumor progression^74,75^. ITIH1 was also specifically shown to inhibit the metastatic process in a human lung cancer cell line^76^.

When the set of 313 unique HPDE vesicle proteins were analyzed for over-represented Reactome pathways, by far the largest number of over-represented pathways grouped to immune system pathways. Twenty-four unique immune system pathways were found to be enriched in normal cell sEV proteins as opposed to only five enriched immune system pathways that were identified in the 348 unique cancer cell sEV proteins (Fig. 5). This is again consistent with the unique presence of immune response proteins in HPDE vesicles. Additionally, a number of gene expression (transcription) pathways were over-represented based on the presence of four DNA-dependent RNA polymerase proteins (POLR3B, POLR3A, POLR3D, and POLR3E) (Fig. 5, Supplementary Data S1). All four proteins are core components of RNA polymerase III and are also involved in immune response^77^. Interestingly, the formation of pre-metastatic niches are contingent on suppression of the immune system^78–80^. Malignant tumor cells can avoid the standard immune response by recruiting myeloid cells for protection, allowing cancer cells to metastasize to other sites^78,81^. These immune response proteins found uniquely in normal pancreatic cells may support a functioning immune response system in a normal cell line that is not observed in the pancreatic cancer cell lines studied here. It will be of interest in the future to study the proteomes of vesicles from other normal pancreatic cell lines to see if this is a common feature of normal healthy cells from different origins.

### Comparison of crude versus sucrose density gradient purified sEVs

**Figure 6:**
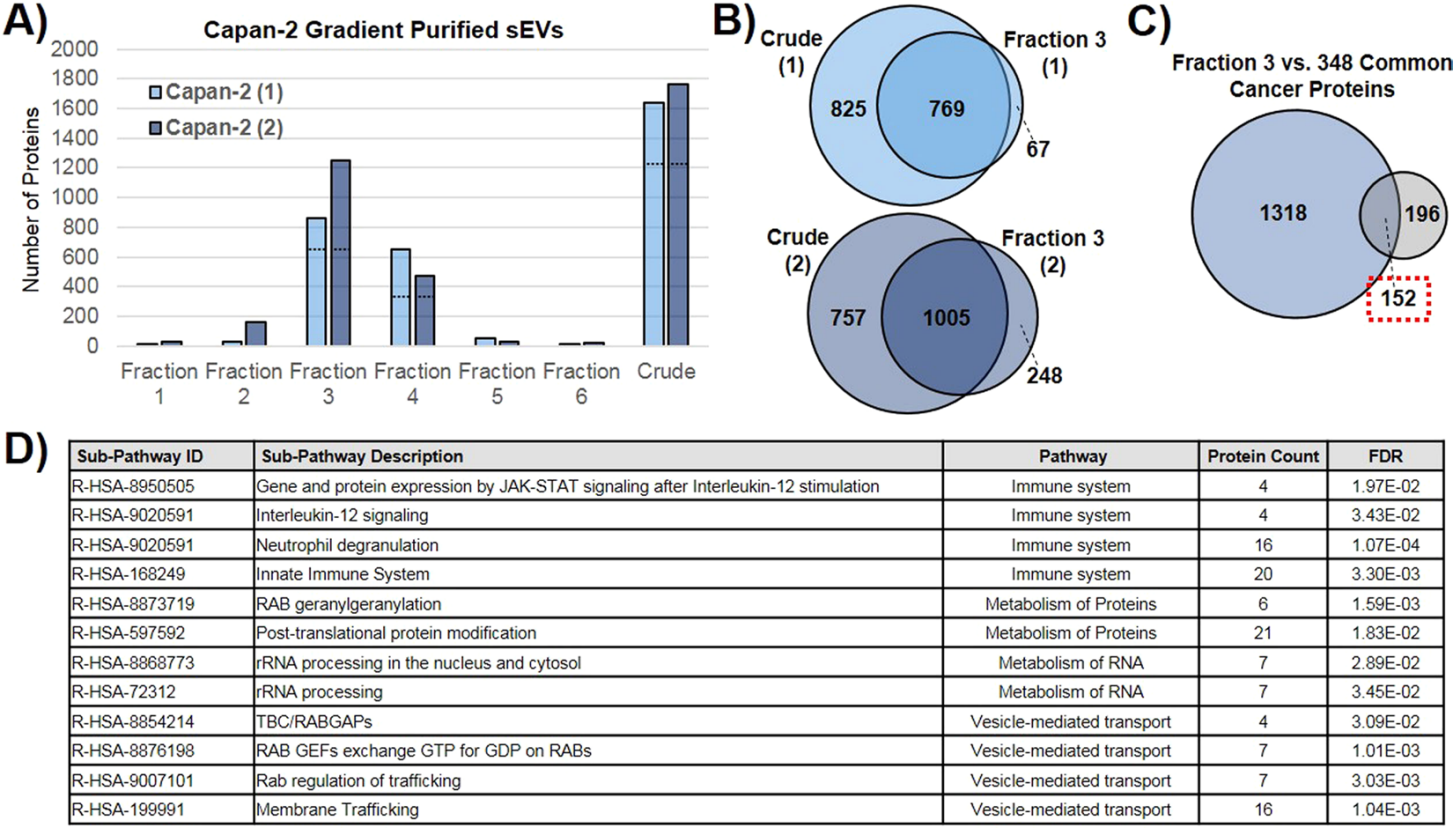
Characterization of Capan-2 sEVs after sucrose density gradient purification. **(A)** Number of proteins found in Fractions 1–6 and the corresponding Crude population of sEVs, two biological replicates shown. The dashed black lines shown on Fraction 3, Fraction 4, and Crude represent the number of proteins found in common between the two biological replicates for each corresponding sample. Crude sEVs were isolated using the ultrafiltration-ultracentrifugation method and then further purified using a sucrose density gradient to produce the six fractions. **(B)** Venn diagrams show the overlap of proteins found in the crude sEVs with the corresponding Fraction 3 samples containing purified sEVs. **(C)** Proteins identified in Fraction 3 Capan-2 sEVs are compared with the 348 common cancer sEV proteins that were analyzed in Fig. 4. **(D)** Significantly enriched Reactome Pathways associated with the 152 proteins found in common between Fraction 3 samples and the common cancer proteins (red dashed box). Enriched Reactome pathways were identified via GO enrichment analysis.

As mentioned previously, crude sEVs analyzed here were isolated using a combined ultrafiltration-ultracentrifugation method to remain consistent with vesicles analyzed previously that were shown to function as initiators of cell transformation^24^. Since it has recently been shown that incorporating a sucrose density gradient purification method into the isolation protocol results in a “more pure” population of sEVs^4^, this was tested with crude Capan-2 sEVs. Two separate biological replicates of crude Capan-2 sEVs were floated onto a sucrose density gradient and six fractions were collected. Each fraction along with the corresponding crude sample was run on SDS-PAGE gel for LC-MS/MS analysis. As expected, the greatest number of proteins were found in Fraction 3 (density of 1.15–1.174 g/mL) for both replicates (Fig. 6A). The Capan-2 purified exosomes were characterized according to guidelines laid out in the MISEV2018^4^. Common sEV marker proteins were identified in the crude isolate, Fraction 3, and Fraction 4 samples by western blot analysis and appear to be enriched in Fractions 3 and 4 relative to the Crude isolate (Supplementary Fig. S1). The size of Fraction 3 Capan-2 sEVs, as determined by nanoparticle tracking analysis, decreased relative to crude sEVs and was centered on 67 nm, while the mean size of particles decreased from 250.3 nm to 83.5 nm (Supplementary Fig. S1, image reprinted with permission from ref. ^24^). Additionally, the measured particles/ug for Fraction 3 Capan-2 sEVs was 4.22 × 10^9^ particles/µg compared to 8.75 × 10^8^ particles/µg for crude sEVs, indicating enhanced purity of sucrose density gradient sEVs^82^. The “top twenty” sEV marker proteins characterized in Fig. 1 were also assessed here to determine the average PSMs found for each protein in the Fraction 3 samples compared to the crude samples (Supplementary Fig. S1). While all twenty proteins were still identified by MS in the purified Fraction 3 sEVs, the number of PSMs observed for each protein has decreased relative to the crude sample, suggesting that overall protein recovery was sacrificed in order to benefit from the enhanced purification of sEVs using sucrose density gradient separation. This decrease in recovery is also apparent when you compare the overlap between proteins found in crude sEVs with those found in Fraction 3 (Fig. 6B). In order to assess whether or not enrichment of sEV marker proteins was achieved by sucrose density purification, the crude Capan-2 sEVs were normalized to the protein concentration of the Fraction 3 sEVs. MS/MS results from ‘Normalized Crude’ sEVs show that a number of sEV marker proteins appear to be enriched in Fraction 3 samples as evidenced by the increase in the number of PSMs observed. Additionally, there were a small percentage of “new” proteins identified in Fraction 3 samples that were not found in crude samples. These likely represent lower abundance proteins that were not detected in Capan-2 crude samples by MS due to sampling limitations.

Previous work showed that Fraction 3 sEVs from Capan-2 cells could initiate malignant cell transformation^24^, so we compared the total proteins identified in Capan-2 Fraction 3 against the 348 unique cancer sEV proteins for overlap (Fig. 6C). Results showed that 152 of the common cancer sEV proteins were still present in Fraction 3 samples after purification. While crude sEVs were only purified for one type of cancer cell (Capan-2), this comparison narrowed down the number of potentially important proteins found to be unique to cancer cell sEVs and not present in normal cell sEVs. GO enrichment analysis for over-represented pathways was carried out on these 152 proteins (Fig. 6D, Supplementary Data S1). Results show that Reactome pathways related to vesicle-mediated transport, metabolism, and immune system were still enriched in the reduced set of 152 proteins. Of these proteins, seven Ras-related proteins were still found, contributing to an observed enrichment in the GO biological process of Ras protein signal transduction (Supplementary Table S4). Ultimately, additional purification of sEVs resulted in a decrease in protein recovery as evidenced by the reduction of proteins found in common between all cancer cells but absent from normal cells. While our work here focused on the proteomic analysis of crude cancer cell sEVs with a previously described phenotype, the use of enhanced purification methods, like the one described here, is now widely expected for studies focused on elucidating the biological function(s) of a particular subtype of extracellular vesicles.

## Conclusion

Our previous results showed that pancreatic cancer cell sEVs could initiate malignant cell transformation while normal cell sEVs could not^24^. In-depth proteomic analysis of those sEVs revealed both commonalities and distinct differences between vesicles secreted from normal and cancer pancreatic cells. The proteins present in all four sEV types were made up of general housekeeping proteins that regulate normal cell functions (Fig. 3). However, cancer cell sEVs contained unique endosomal proteins that play different roles in the formation and secretion of sEVs from cells (Fig. 4A). Secretion of these vesicles may be critical for cancer progression, as a growing number of studies have highlighted the contributions of cancer cell-derived sEVs to cell-cell communication and metastasis, specifically in the formation of pre-metastatic niches^13,15^. Also, all three cancer cell sEVs contained specific proteins that have been shown to contribute to malignant cell transformation, consistent with our previous result^24^. Interestingly, sEVs derived from normal pancreatic cells were enriched for immune response proteins that were lacking in cancer cell vesicles (Fig. 4B). Suppression of the immune system also plays a large role in cancer progression, as it allows for the formation of pre-metastatic niches^78,79^. These unique differences found between the proteins packaged in cancer and normal cell sEVs may point towards their potential value as cancer biomarkers.

## Methods

### Cell culture and isolation of sEVs

Human pancreatic cancer cell lines (Capan-2, MIA PaCa-2, and Panc-1) were purchased from American Type Culture Collection (Manassas, VA). The immortalized human pancreatic duct epithelial (HPDE) cell line was purchased from Kerafast (Boston, MA) and these cells demonstrate near normal genotype and phenotype of HPDE cells and are morphologically similar to the primary HPDE cells. Capan-2, MIA PaCa-2, and Panc-1 cells were maintained in Dulbecco’s modified Eagle’s medium (DMEM) (Millipore Sigma) supplemented with 10% (v/v) fetal bovine serum (Millipore Sigma) and 1% (v/v) antibiotics solution (Penicillin-Streptomycin, Millipore Sigma). HPDE cells were maintained in Keratinocyte Serum-Free Media (KSFM) (Invitrogen) with KSFM Supplements including epithermal growth factor (EGF) and bovine pituitary extract (BPE) (Invitrogen). All cell lines were cultured at 37 °C in a humidified atmosphere of 5% CO_2_. Each cell line was tested free from mycoplasma. HPDE cells were used below passage 8 (p < 8) and the three cancer cell types were used below passage 20 (p < 20).

All sEVs were isolated from cells using a previously described combined ultrafiltration-ultracentrifugation protocol^24,27^. Pancreatic cancer cells (Capan-2, MIA PaCa-2, and Panc-1) and normal pancreatic cells (HPDE) were grown in ten 225 cm^3^ flasks in standard medium until they reached a confluency of ~70–80% (~3.5 × 10^8^ cells). Pancreatic cancer cell lines were washed twice with medium before being incubated in plain, serum-free medium for 72 hr. HPDE cells were washed with phosphate-buffered saline (PBS) and incubated in plain KSFM medium without supplements for 72 hr. This protocol did not measurably increase the rate of cell death as determined by trypan blue exclusion, which showed over 93% live cell counts after 72 hr incubation in conditioned media. Next, the conditioned media (~450 mL) from serum-free cell cultures containing sEVs were cooled down on ice, centrifuged (350xg, 10 min) (Beckman JA-10 rotor, k-Factor = 3,610), and passed through a 0.2 μm pore filter to remove cells, cellular debris, and vesicles sized larger than 220 nm. An inhibitor cocktail was added to protect the proteins from proteolytic digestion (PMSF and inhibitor cocktail complete Roche, Mannheim, Germany). Enrichment of sEVs was carried out by subsequent ultrafiltration with an Amicon Ultra 100 K filter (4000×g, 25 min, 4 °C) (Beckman SX4750 rotor, k-Factor = 13,458.8) and ultracentrifugation (120,000×g, 90 min, 4 °C) (Beckman TLA-110 rotor, k-Factor = 13). The sEV pellets were washed in PBS and then ultracentrifuged again (120,000xg, 90 min, 4 °C). The final sEV pellets were resuspended in PBS and validated by characterization via MS analysis of proteins, western blot analysis, electron microscopy analysis (TEM), and nanoparticle tracking analysis (NTA)^4,24^. The protein concentration of sEVs was measured after each isolation using the CBQCA protein quantitation kit (Invitrogen) and normalized across all crude samples before MS analysis. The k-Factor for the Beckman SX4750 rotor was not available and was calculated manually via https://www.beckman.com/centrifuges/rotors/calculator.

The antibodies used for western blot analyses are as follows: CD63 (rabbit polyclonal) RRID:AB_2783831, Anti-ALIX (3A9) (mouse monoclonal) RRID:AB_10899268, TSG101 (4A10) (mouse monoclonal) RRID:AB_2208088, Anti-β-actin (AC-74) (mouse monoclonal) RRID: AB_476697, HSP90α/β (F8) (mouse monoclonal) RRID:AB_675659, Calnexin (C5C9) (rabbit monoclonal) RRID:AB_2228381, α-actinin (H-2) (mouse monoclonal) RRID:AB_626633, C1orf116 antibody, specifically androgen-regulated gene protein (SARG) (rabbit polyclonal), RRID: AB_2228225, ALDH1A1 (clone#1A10A2) (mouse monoclonal) RRID: AB_10693634, CD13 (clone# 2D8D11) (mouse monoclonal) Proteintech (cat# 66211-1-Ig), and RHOB (rabbit polyclonal) RRID: AB_2179092.

### Sucrose density gradient purification

Two biological replicates of crude Capan-2 sEVs isolated using the protocol described above were further purified via floatation onto a sucrose density gradient. Sucrose gradients were built manually as described previously by first preparing 12 sucrose stock fractions in PBS, concentrations ranging from 10–90%^7,24,28^. Half of the crude sEV pellet from the isolation protocol described above was resuspended in 50 µl PBS with 1 ml of 90% sucrose stock solution and loaded at the bottom of a 13.2 mL ultra-clear Beckman ultracentrifuge tube. The gradient was layered by sequentially pouring 1 mL of each of the remaining 11 solutions in order from highest to lowest sucrose concentration. Tubes were centrifuged in a TH-641 rotor (Thermo) (100,000xg, 16 hr, 4 °C) (24200 rpm, k-Factor = 114). At completion, six 2 mL fractions were collected from each of the tubes. Next, 9 mL of PBS was added to each of the six fractions and centrifuged (100,000xg, 1 hr, 4 °C). The resulting pellets were resuspended in 50 µl PBS and validated by mass spectrometry, western blot, and nanoparticle tracking analysis (NTA)^24^. The densities of the six fractions were previously determined and are as follows: Fraction 1 (1.076–1.088 g/mL), Fraction 2 (1.108–1.126 g/mL), Fraction 3 (1.15–1.174 g/mL), Fraction 4 (1.195–1.216 g/mL), Fraction 5 (1.243–1.268 g/mL), and Fraction 6 (1.287–1.290 g/mL). The protein concentration of the six fractions were measured using a CBQCA protein quantitation kit (Invitrogen). Particle size was determined via NTA for crude Capan-2 sEVs (8.75 × 10^8^ particles/µg) and Fraction 3 purified Capan-2 sEVs (4.22 × 10^9^ particles/µg).

### Proteomic mass spectrometry analysis

For analysis of sEV proteins by mass spectrometry, sample preparation included the excision of proteins from polyacrylamide gels via SDS-PAGE. Approximately 15 μg of protein from sEVs isolated from each cell line were thawed from −80 °C storage and combined with 10 μl of 5x protein sample buffer. Samples were run ~10 mm into the top of a TGX stain‐free gel (Bio‐Rad) before the total gel band containing proteins was excised. Protein samples were reduced and alkylated using DTT and iodoacetamide, respectively. Samples were digested overnight using trypsin (37 °C) and resulting peptides were de-salted using solid phase extraction (SPE). LC-MS/MS experiments were performed on a Thermo Scientific EASY-nLC 1200 liquid chromatography system coupled to a Thermo Scientific Orbitrap Fusion Lumos mass spectrometer. To generate MS/MS spectra, MS1 spectra were first acquired in the Orbitrap mass analyzer (resolution 120,000). Peptide precursor ions were then isolated and fragmented using high-energy collision-induced dissociation (HCD). The resulting MS/MS fragmentation spectra were acquired in the ion trap. MS/MS spectral data from samples was searched using Proteome Discoverer 2.1 software (Thermo Scientific) against entries included in the Human Uniprot protein database (70,451 entries, Proteome ID = IP000005640). Search parameters included Carbamidomethylation of cysteine residues (+57.021 Da) as a static modification and oxidation of methionine (+15.995 Da) and acetylation of peptide N-termini (+42.011 Da) as dynamic modifications. The precursor ion mass tolerance was set to 10 ppm and the product ion mass tolerance was set to 0.6 Da for all searches. Peptide spectral matches were adjusted to a 1% false discovery rate (FDR) and proteins were filtered to a 1% FDR. For crude sEV samples (Capan-2, MIA PaCa-2, Panc-1, and HPDE), results from three biological replicates were combined. For sucrose density gradient purified Capan-2 sEVs, two separate biological replicates of Fraction 3 separated vesicles were compared to their corresponding crude sample. For the ‘Normalized Crude’ Capan-2 sample, protein concentration was normalized to the corresponding Fraction 3 sample before MS/MS analysis was performed. Due to sample limitations, only a single biological replicate was run for the normalized crude sample only. A complete list of filtered proteins identified in crude Capan-2, MIA PaCa-2, Panc-1, and HPDE sEVs can be found as Supplementary Data S2 online. A complete list of filtered proteins identified in each Capan-2 Crude and Fraction 3 sample can be found as Supplementary Data S3 online. Gene ontology (GO) enrichment analysis and Reactome pathway analysis of protein datasets was performed via PANTHER 14.1^30,32,33^.

## Supplementary information

Supplementary Information.

Supplementary Data S1.

Supplementary Data S2.

Supplementary Data S3.
