# Supplementary material for: Proteomic Profiling of Small Extracellular Vesicles Secreted by Human Pancreatic Cancer Cells Implicated in Cellular Transformation
Source: Sci Rep. 2020 May 7;10:7713. doi: 10.1038/s41598-020-64718-6 (PMC7205864; doi:10.1038/s41598-020-64718-6)
Supplement: Supplementary file 1 — Supplementary Information. [file 41598_2020_64718_MOESM1_ESM.docx]

**Proteomic Profiling of Small Extracellular Vesicles Secreted by Human Pancreatic Cancer Cells Implicated in Cellular Transformation**

Kelly A. Servage^1,2#^, Karoliina Stefanius^1,2#^, Hillery Fields Gray^1,2^, and Kim Orth^1,2*^

^1^Department of Molecular Biology, University of Texas Southwestern Medical Center, Dallas, TX, USA

^2^Howard Hughes Medical Institute, University of Texas Southwestern Medical Center, Dallas, TX, USA

# These authors contributed equally.

*Correspondence: Kim Orth ([Kim.Orth@UTSouthwestern.edu](mailto:Kim.Orth@UTSouthwestern.edu))

**Supplementary Figures:**

**
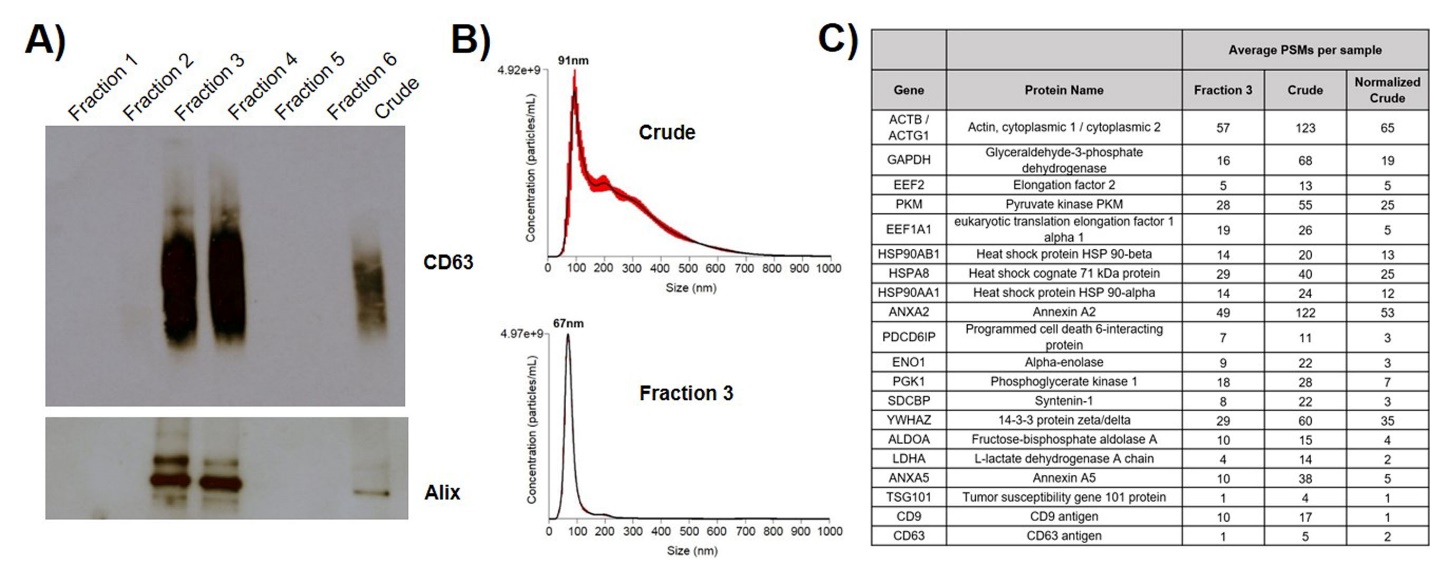
**

**Supplementary Figure S1.** Characterization of Capan-2 sEVs after sucrose density gradient purification. **A)** Western blot analysis of common sEV marker proteins CD63 and Alix in Capan-2 sEVs. Crude sEVs were isolated using the ultrafiltration-ultracentrifugation method and then further purified using a sucrose density gradient to produce the six fractions. **B)** Nanoparticle tracking analysis of crude sEVs and Fraction 3 (purified) sEVs from Capan-2 cells. Data represent average size per concentration (black line) ± standard error of the mean (red bars) of three measurements from one sEV preparation. For the crude sEVs, vesicle size is centered on 91 nm with a mean size of 250.3 nm (8.75x10^8^ particles/µg). For Fraction 3 sEVs, vesicle size is centered on 67 nm with a mean size of 83.5 nm (4.22x10^9^ particles/µg). Finite Track Length Analysis (FTLA) was used for size determination (reprinted from Ref. 24). **C)** Table of common sEV marker proteins found in crude and Fraction 3 samples shown in Figure 6 (labeled Fraction 3 and Crude): average peptide spectral matches (PSMs) were calculated from two biological replicates for both Fraction 3 purified sEVs and crude Capan-2 sEVs. The final column in the table labeled Normalized Crude represents the number of PSMs found after normalizing the protein concentration of Crude Capan-2 sEVs to the corresponding Fraction 3 sample; results represent a single biological replicate.

**
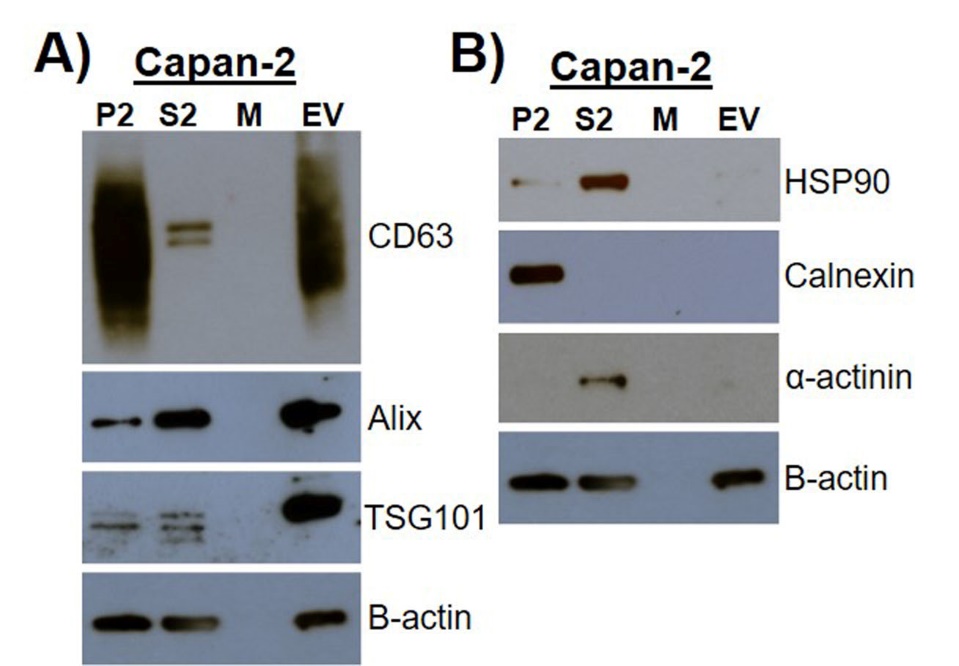
**

**Supplementary Figure S2. A)** Western blot analysis of common sEV marker proteins CD63, Alix, and TSG10 found in sEVs isolated from Capan-2 cells. **B)** Western blot analysis of proteins HSP90, Calnexin, and α-actinin, expected to be underrepresented in sEVs. B-actin is used as a loading control. Equivalent amounts of proteins from ER and mitochondria (P2), cytoplasm (S2), media (M), and crude sEV (EV) fractions derived from the Capan-2 sEV isolation process were analyzed (reprinted from Ref. 24).

**
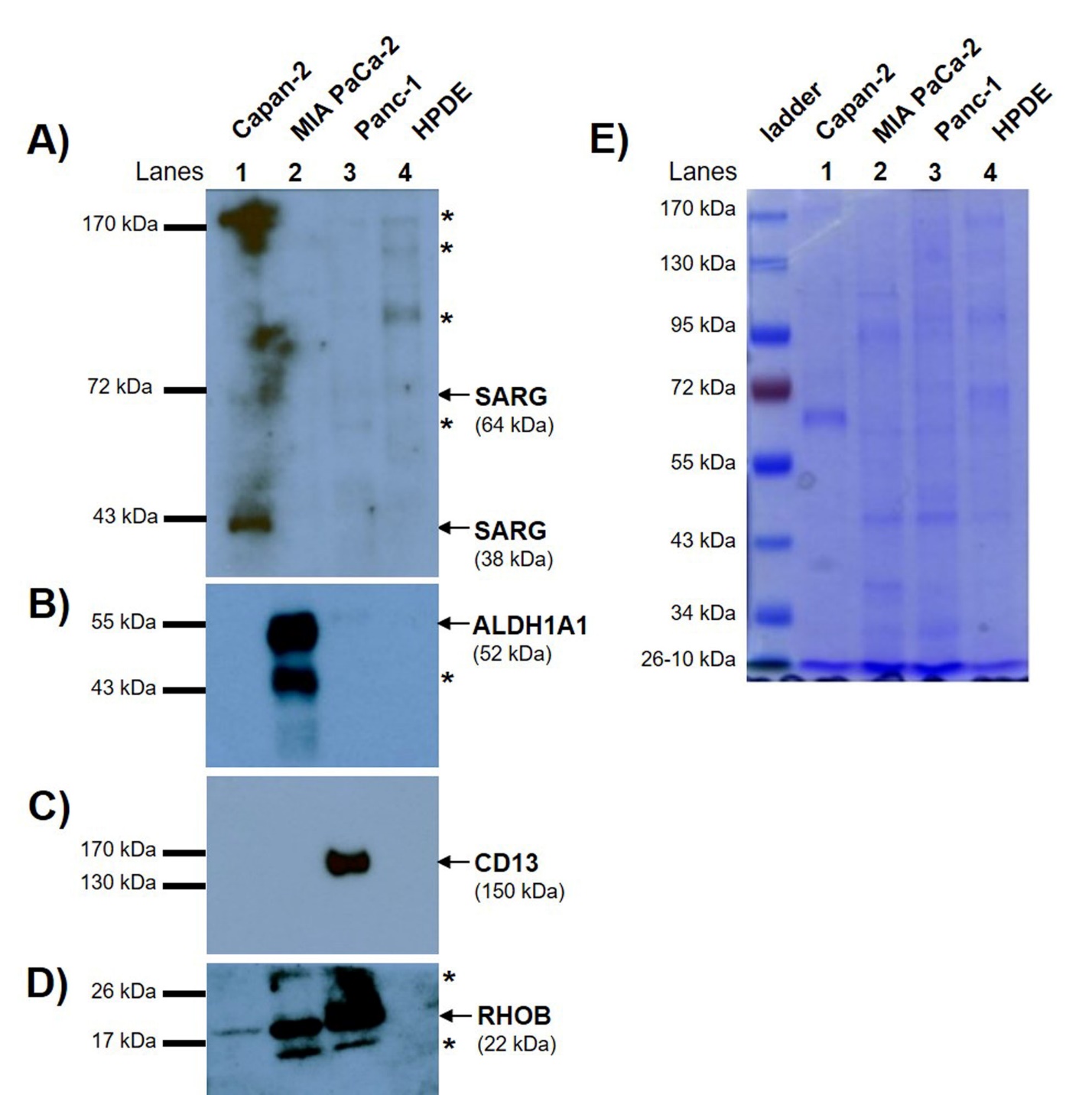
**

**Supplementary Figure S3.** Western blot analysis of representative proteins found by MS/MS uniquely in only one type of sEV (**A-C**) or found in the group of 348 common cancer sEV proteins (**D**). Specifically (**A**) androgen-regulated gene protein (SARG), (**B**) Aldehyde dehydrogenase family 1 member A1 (ALDH1A1), (**C**) Aminopeptidase N (CD13), and (**D**) Ras homolog gene family, member B (RHOB). (**E**) Coomassie staining as a loading control for Western blot analysis. Labeled arrows indicate the location of expected bands with the observed molecular weight (in parenthesis) according to antibody vendors. Asterisks indicate background bands.

**
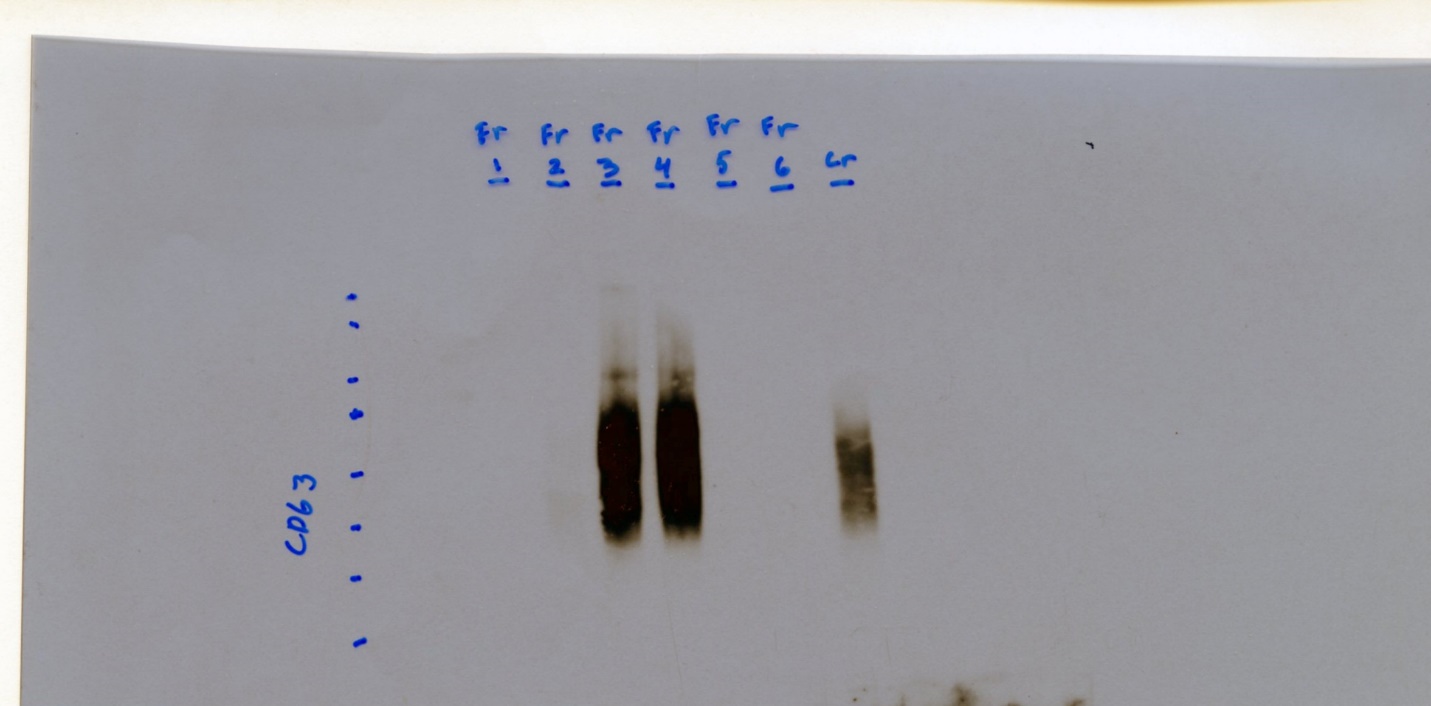
**

**Supplementary Figure S4.** Original image of western blot for CD63 as shown in Supplementary Figure S1A. Antibody used: CD63 (rabbit polyclonal), RRID:AB_2783831.

**
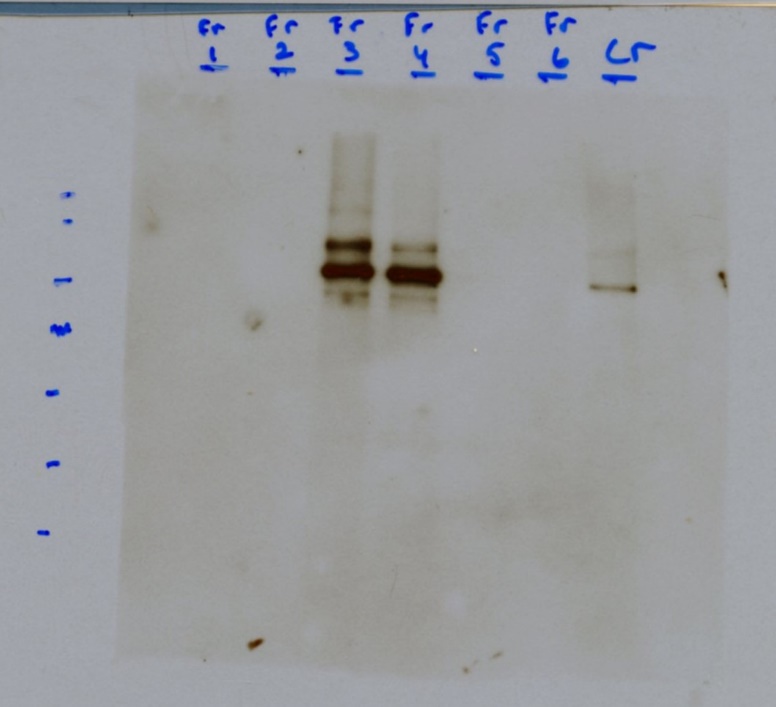
.**

**Supplementary Figure S5.** Original image of western blot for Alix as shown in Supplementary Figure S1A. Antibody used: Anti-ALIX (3A9) (mouse monoclonal), RRID:AB_10899268.

**
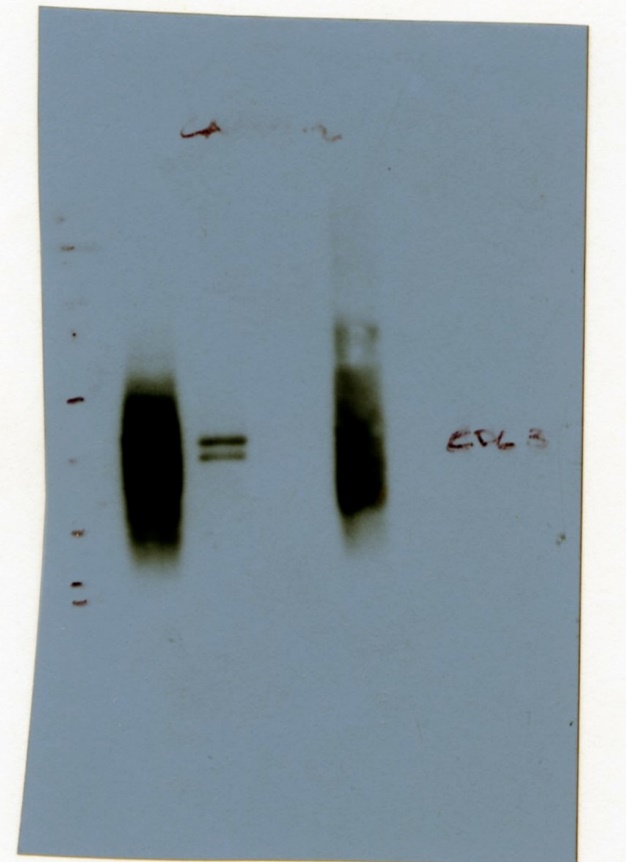
**

**Supplementary Figure S6.** Original image of western blot for CD63 as shown in Supplementary Figure S2A. Antibody used: CD63 (rabbit polyclonal), RRID:AB_2783831.

**
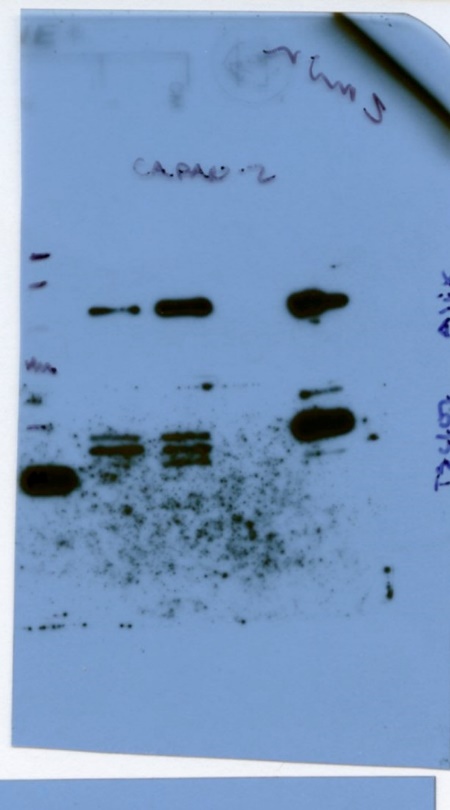
**

**Supplementary Figure S7.** Original image of western blot for Alix (top band) as shown in Supplementary Figure S2A. Antibody used: Anti-ALIX (3A9) (mouse monoclonal), RRID:AB_10899268.

**
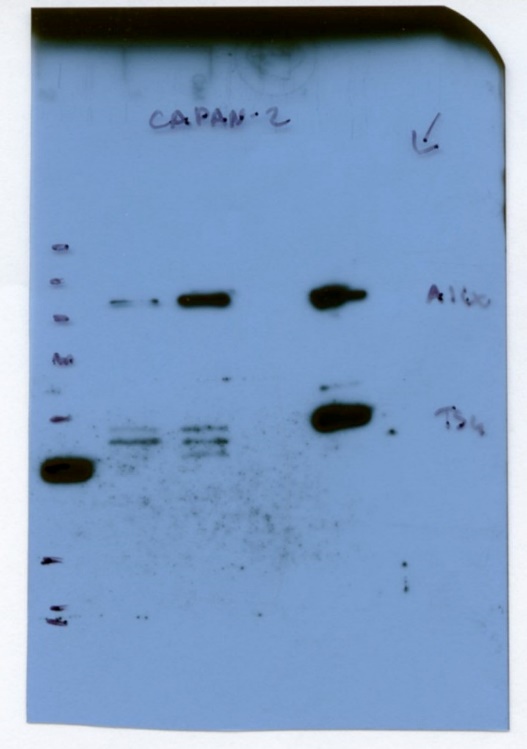
**

**Supplementary Figure S8.** Original image of western blot for TSG101 (bottom band) as shown in Supplementary Figure S2A. Antibody used: TSG101 (4A10) (mouse monoclonal), RRID:AB_2208088.

**
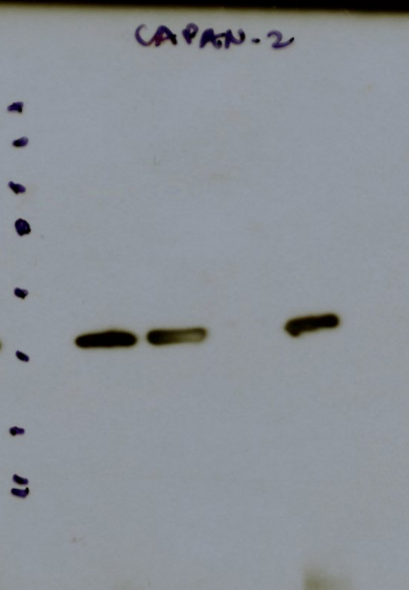
**

**Supplementary Figure S9.** Original image of western blot for B-actin as shown in Supplementary Figure S2. Antibody used: Anti-β-actin (AC-74) (mouse monoclonal), RRID: AB_476697.


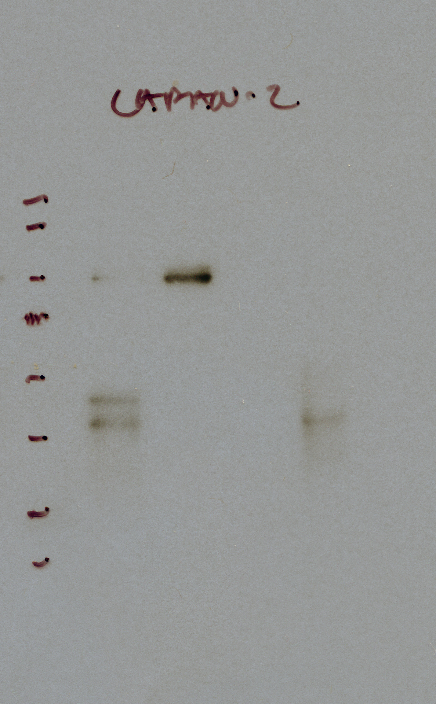


**Supplementary Figure S10.** Original image of western blot for HSP90 as shown in Supplementary Figure S2B. Antibody used: HSP90α/β (F8) (mouse monoclonal), RRID:AB_675659.


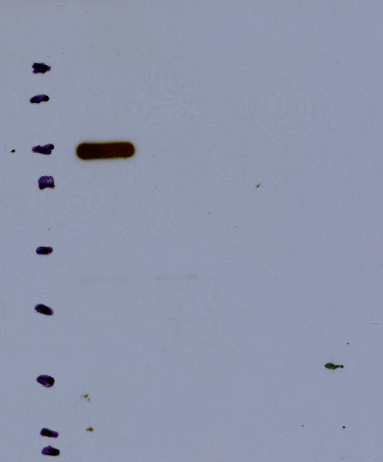


**Supplementary Figure S11.** Original image of western blot for Calnexin as shown in Supplementary Figure S2B. Antibody used: Calnexin (C5C9) (rabbit monoclonal), RRID:AB_2228381.


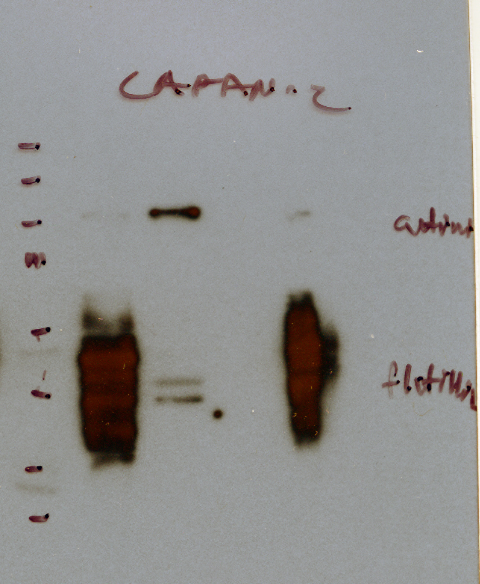


**Supplementary Figure S12.** Original image of western blot for α-actinin (top band) as shown in Supplementary Figure S2B. Antibody used: α-actinin (H-2) (mouse monoclonal), RRID:AB_626633.


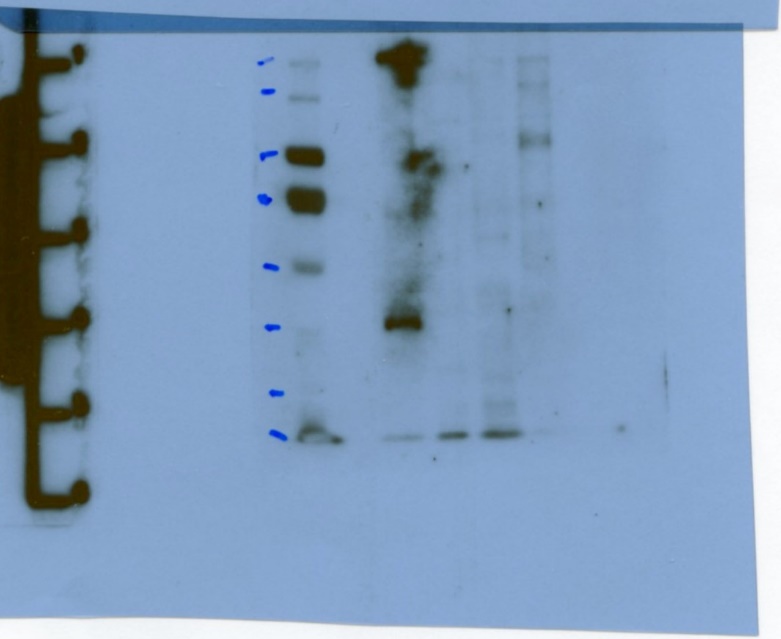


**Supplementary Figure S13.** Original image of western blot for SARG as shown in Supplementary Figure S3A. Antibody used: C1orf116 antibody, specifically androgen-regulated gene protein (SARG) (rabbit polyclonal), RRID: AB_2228225.


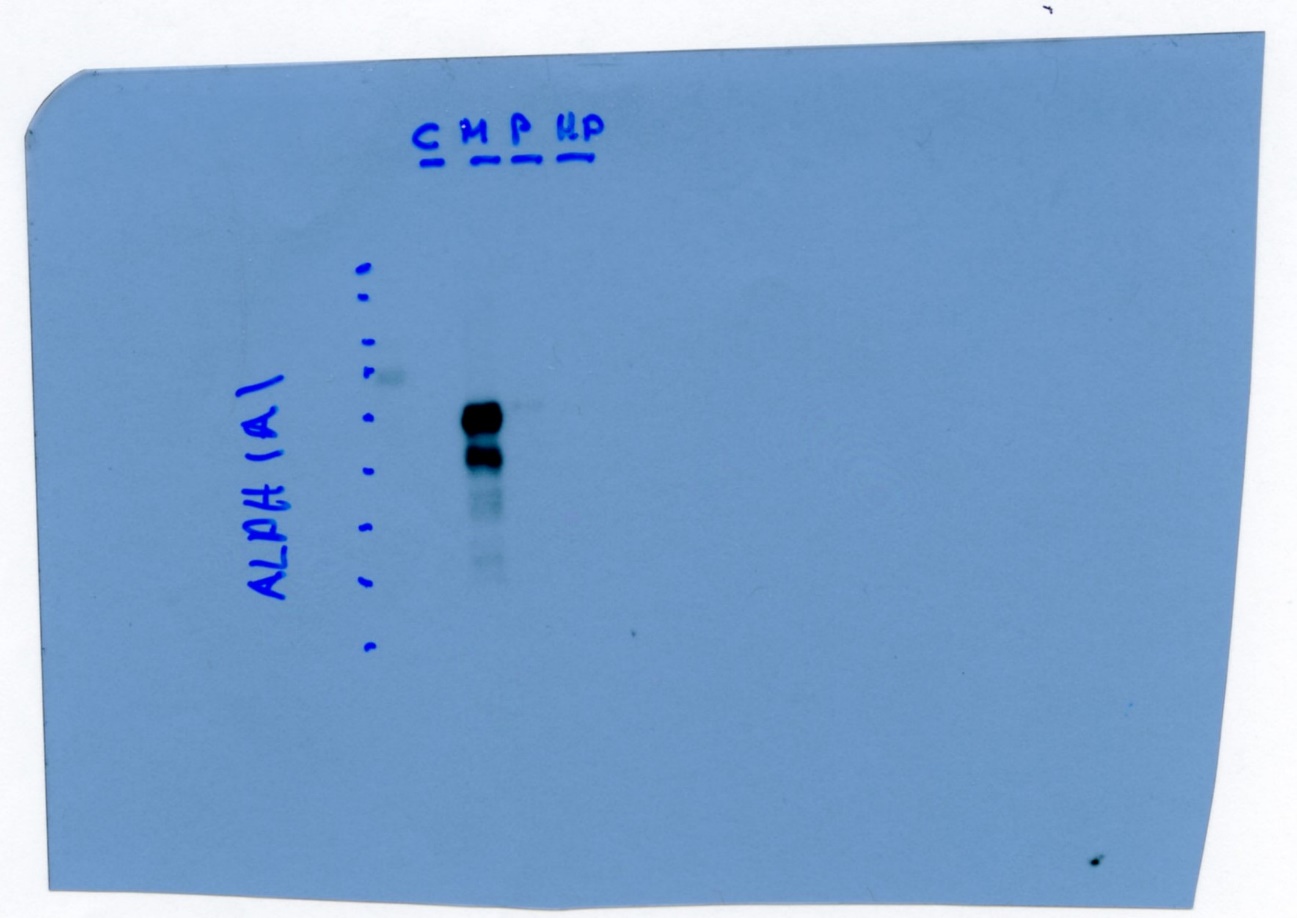


**Supplementary Figure S14.** Original image of western blot for ALDH1 as shown in Supplementary Figure S3B. Antibody used: ALDH1A1 (clone#1A10A2) (mouse monoclonal), RRID: AB_10693634.


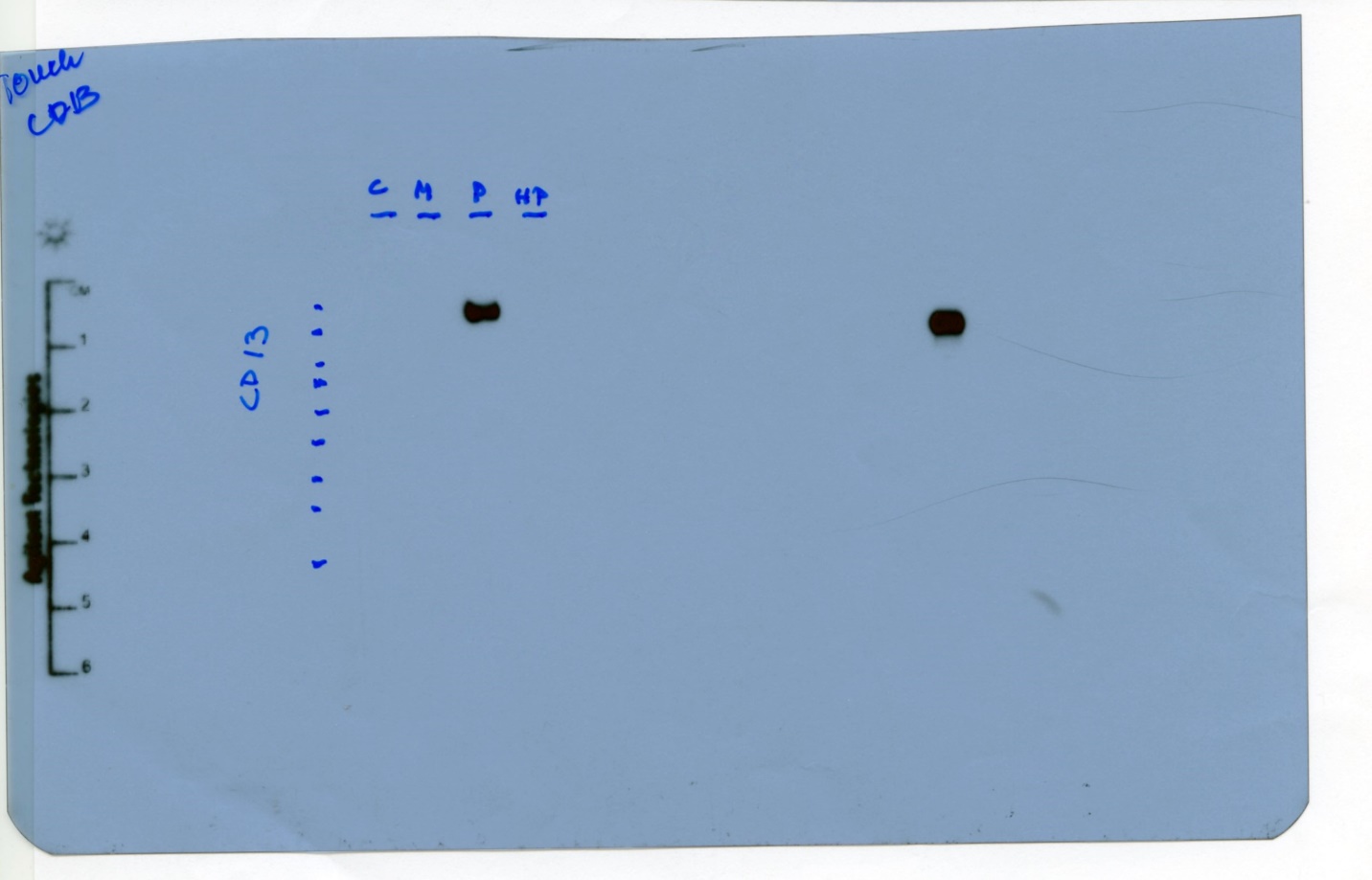


**Supplementary Figure S15.** Original image of western blot for CD13 as shown in Supplementary Figure S3C. Antibody used: CD13 (clone# 2D8D11) (mouse monoclonal), Proteintech (cat# 66211-1-Ig).


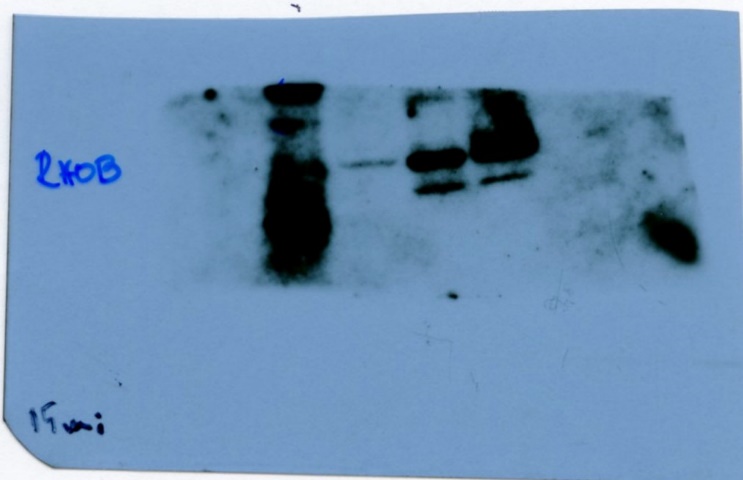


**Supplementary Figure S16.** Original image of western blot for RHOB as shown in Supplementary Figure S3A. Antibody used: RHOB (rabbit polyclonal), RRID: AB_2179092.

**Supplementary Tables:**


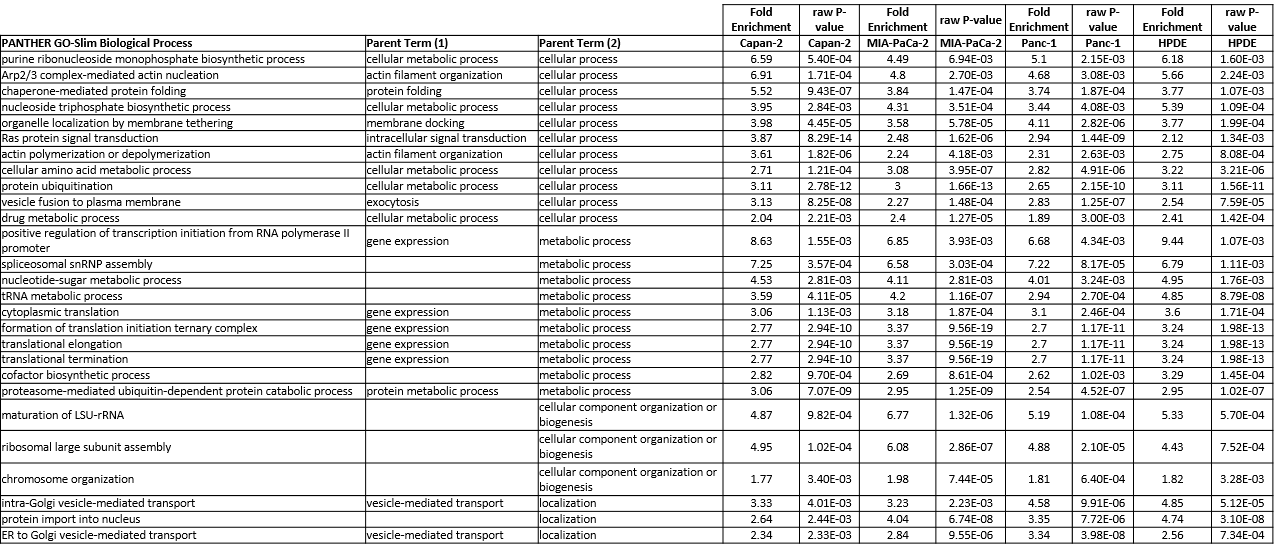


**Supplementary Table S1.** Enriched biological processes shown in **Fig. 3** graph along with the parent terms associated with each biological process. Parent terms were identified using PANTHER 14.1. The fold enrichment score and raw P-value for each enriched process was found by GO enrichment analysis via PANTHER 14.1. P-values < 0.05 were considered as statistically significant.


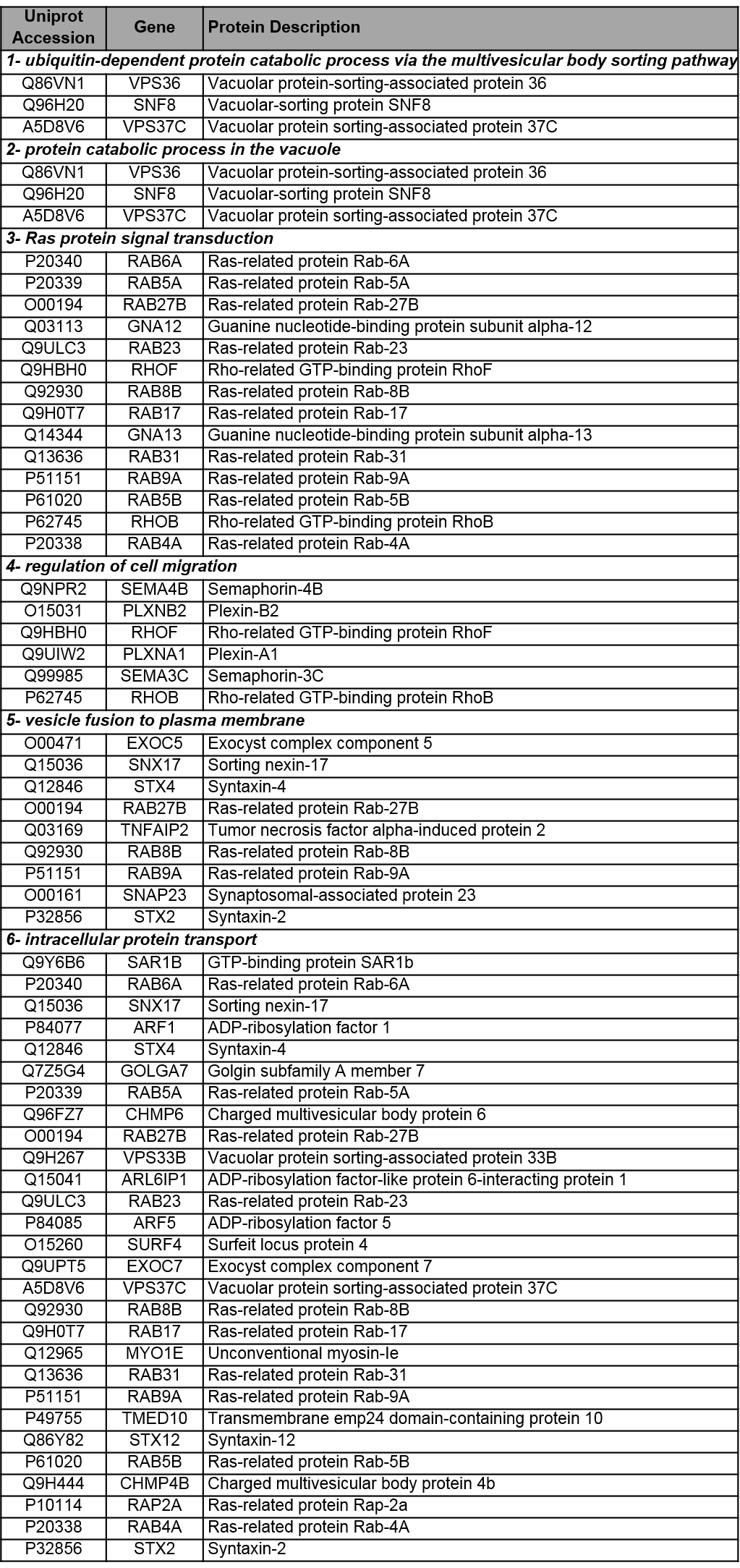


**Supplementary Table S2.** Proteins associated with each GO biological process found to be enriched in the set of 348 common pancreatic cancer sEV proteins shown in **Fig. 4A**.


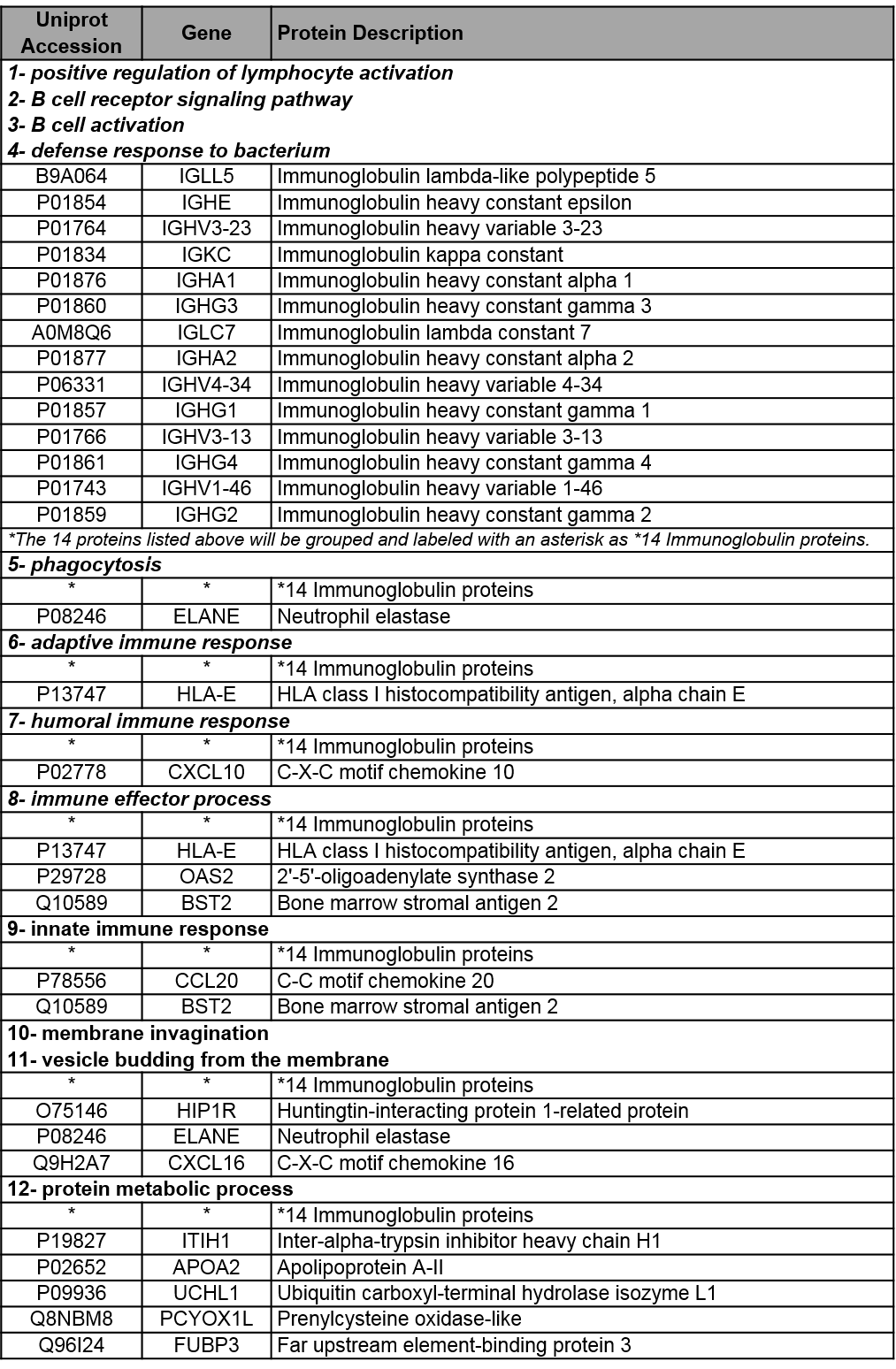


**Supplementary Table S3.** Proteins associated with each GO biological process found to be enriched in the 313 unique HPDE proteins shown in **Fig. 4B**.


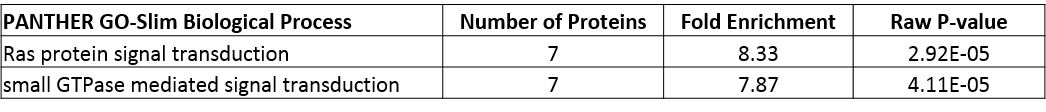


**Supplementary Table S4.** Enriched biological processes found after GO enrichment analysis (PANTHER 14.1) on the 152 proteins shown in **Fig. 5** (overlap between Fraction 3 proteins and 348 common cancer proteins). P-values < 0.05 were considered as statistically significant.

**Supplementary Data:**

**Supplementary Data S1** (xlsx file) contains a full list of proteins associated with Reactome pathways listed in **Fig. 5** and **Fig. 6**.

**Supplementary Data S2** (xlsx file) contains a complete list of filtered proteins identified in crude Capan-2, MIA PaCa-2, Panc-1, and HPDE sEVs.

**Supplementary Data S3** (xlsx file) contains a complete list of filtered proteins identified in each Capan-2 Crude and Fraction 3 sample.
